# *Toxoplasma gondii* rhoptry discharge factor 3 is essential for invasion and microtubule-associated vesicle biogenesis

**DOI:** 10.1371/journal.pbio.3002745

**Published:** 2024-08-13

**Authors:** Rouaa Ben Chaabene, Matthew Martinez, Alessandro Bonavoglia, Bohumil Maco, Yi-Wei Chang, Gaëlle Lentini, Dominique Soldati-Favre

**Affiliations:** 1 Department of Microbiology and Molecular Medicine, Faculty of Medicine, University of Geneva, Geneva, Switzerland; 2 Department of Biochemistry and Biophysics, Perelman School of Medicine, University of Pennsylvania, Philadelphia, Pennsylvania, United States of America; 3 Institute of Structural Biology, Perelman School of Medicine, University of Pennsylvania, Philadelphia, Pennsylvania, United States of America; Deakin University, Australia, AUSTRALIA

## Abstract

Rhoptries are specialized secretory organelles conserved across the Apicomplexa phylum, essential for host cell invasion and critical for subverting of host cellular and immune functions. They contain proteins and membranous materials injected directly into the host cells, participating in parasitophorous vacuole formation. *Toxoplasma gondii* tachyzoites harbor 8 to 12 rhoptries, 2 of which are docked to an apical vesicle (AV), a central element associated with a rhoptry secretory apparatus prior to injection into the host cell. This parasite is also equipped with 5 to 6 microtubule-associated vesicles, presumably serving as AV replenishment for iterative rhoptry discharge. Here, we characterized a rhoptry protein, rhoptry discharge factor 3 (RDF3), crucial for rhoptry discharge and invasion. RDF3 enters the secretory pathway, localizing near the AV and associated with the rhoptry bulb. Upon invasion, RDF3 dynamically delocalizes, suggesting a critical role at the time of rhoptry discharge. Cryo-electron tomography analysis of RDF3-depleted parasites reveals irregularity in microtubule-associated vesicles morphology, presumably impacting on their preparedness to function as an AV. Our findings suggest that RDF3 is priming the microtubule-associated vesicles for rhoptry discharge by a mechanism distinct from the rhoptry secretory apparatus contribution.

## Abstract 2

Regulated secretion of rhoptry factors is essential for host cell invasion by Apicomplexan parasites. This study identifies the protein rhoptry discharge factor 3 (RDF3) of Toxoplasma gondii as crucial for invasion, by priming microtubule-associated vesicles for rhoptry discharge.

## Introduction

The phylum of Apicomplexa comprises a large group of unicellular eukaryotic parasites that are of medical and veterinary importance. *Toxoplasma gondii* is the most ubiquitous member of the phylum with about one third of the human population chronically infected. This opportunistic pathogen is life-threating in immunocompromised individuals [1] and can cause miscarriage, stillbirth, and congenital anomalies in newborns if primary infection occurs during pregnancy.

Host cell invasion is a vital step for the survival and dissemination of this obligate intracellular parasite. Active entry into host cells is a multistep process that occurs in as fast as ~30 s and involves the sequential, regulated secretion of 2 sets of specialized apical secretory organelles called micronemes and rhoptries. Microneme exocytosis is instrumental for parasite egress from infected cells, motility, and invasion, whereas discharge of rhoptries occurs at the time of invasion [2–4]. Rhoptries are club-shaped organelles that secrete RON (rhoptry neck) and ROP (rhoptry bulb) proteins implicated in invasion and subversion of host cellular functions, respectively. Rhoptries are formed de novo during endodyogeny, at a late stage of the division cycle [5]. *T*. *gondii* tachyzoite possesses 8 to 12 rhoptries, and disruption of the cargo receptor, sortilin-like receptor (SORTLR), impairs their biogenesis [6]. The armadillo repeats only protein (ARO) is crucial for the clustering and the apical anchoring of these organelles [7], while the coccidian-specific CORVET/HOPS Associated Protein (CSCHAP) is implicated in the apical positioning only [8]. Conditional depletion of any of these proteins leads to a severe block in invasion resulting from the absence of rhoptry discharge. The 3D structure and organization of the elements surrounding the rhoptries at the conoid have recently been resolved by cryo-electron tomography (cryo-ET) [9–12]. Two rhoptries enter the conoid [13,14] and are aligned along a pair of intraconoidal microtubules (ICMTs) with their necks reaching an apical vesicle (AV) located underneath the plasma membrane at the tip of the parasite. The AV is docked to an elaborate structure called “rhoptry secretion apparatus” (RSA) inserted in the parasite plasma membrane [10,15]. Five to 6 microtubule-associated vesicles (MVs) are aligned along the ICMTs and thought to be involved in replenishing the AV and enabling successive rounds of rhoptry discharge [13,16]. Concordantly, MVs and ICMTs are only present in parasites that harbor more than 2 rhoptries. All these elements are presumably functionally linked to rhoptry discharge in *T*. *gondii* [9,17]. Remarkably, several proteins involved in rhoptry discharge, such as the complex of non-discharge (Nd) proteins and cysteine repeats modular proteins (CRMPs) [9,18], are conserved outside Apicomplexa, in other Alveolate species (e.g., Ciliates) that contain organelles evolutionarily related to rhoptries [19,20]. Two proteins, initially designated as MIC15 and MIC14, have been identified to play a role in rhoptry discharge and were subsequently renamed RDF1 and RDF2, respectively [21]. These proteins exhibit a scattered punctate distribution that does not resemble any known subcellular compartment and have been identified as components of the CRMPs complex [18,22]. Additionally, a rhoptry apical surface protein 2 (RASP2) covers the end of the rhoptry neck and is crucial for rhoptry discharge [23].

While the machinery involved in positioning rhoptries apically and facilitating their discharge has been extensively investigated, there remains a critical knowledge gap regarding how rhoptry content reaches the host cell cytosol. In this study, we present the characterization of rhoptry discharge factor 3 (RDF3), which is essential for both rhoptry discharge and invasion of *T*. *gondii*. RDF3 exhibits a unique dual localization near the AV and associated with the rhoptry bulb, and these localizations undergo dynamic changes during invasion. Cryo-ET analysis of RDF3-depleted parasites, along with mutant phenotype characterization, underscores its crucial role in MV biogenesis and, consequently, in AV function.

## Results

### Identification of rhoptry proteins in *Toxoplasma gondii*

**Fig 1:**
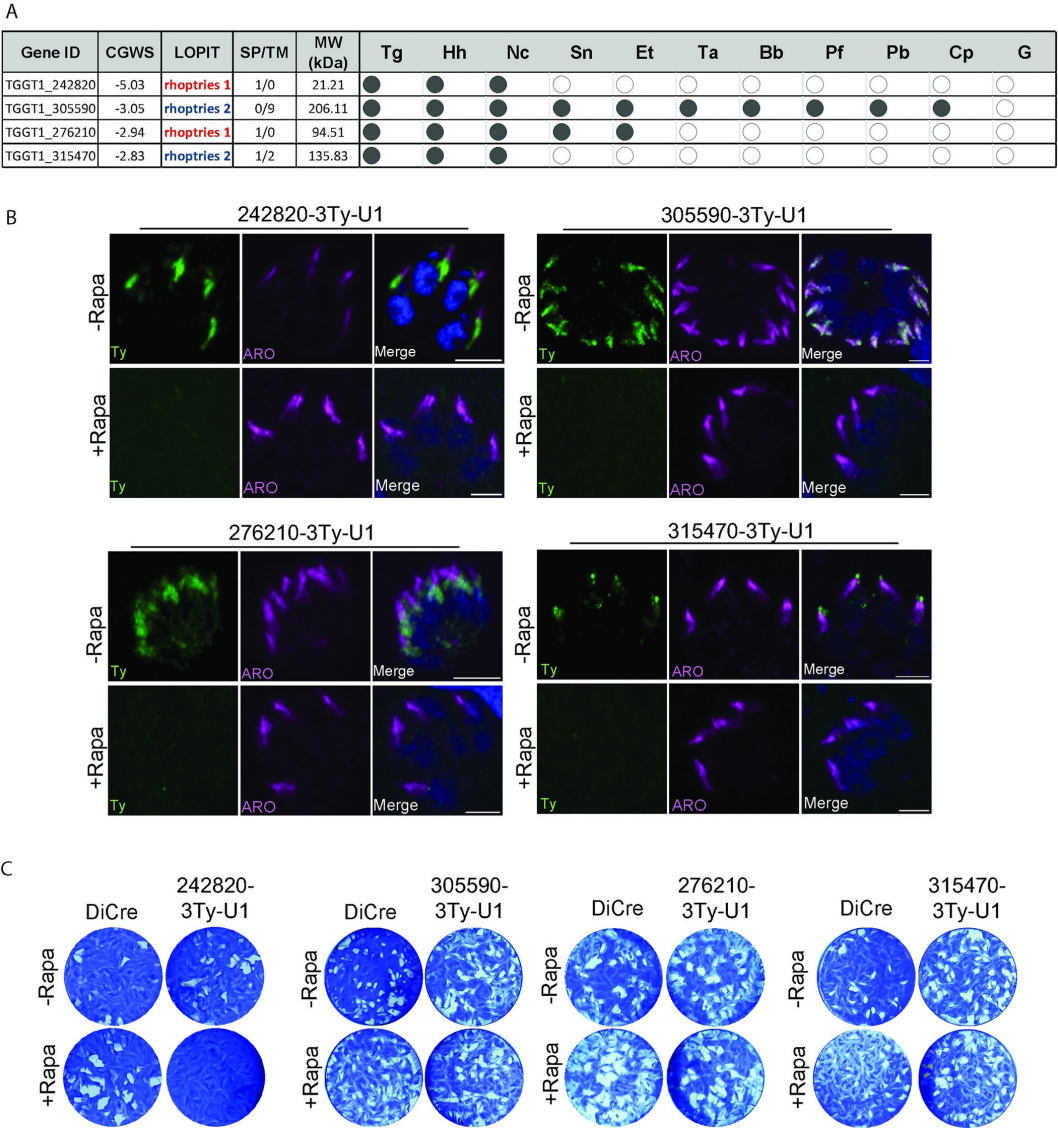
Identification of rhoptry proteins in *Toxoplasma gondii*. (A) Table includes the predicted subcellular localization (LOPIT) [26], the fitness score (CGWS: CRISPR-Cas9 Genome Wide Screen) [24], the number of predicted TMs, and the presence of SP, the molecular weight and conservation across Apicomplexa. *Toxoplasma gondii* (Tg), *Hammondia hammondia* (Hh), *Neospora caninum* (Nc), *Sarcocystis neurona* (Sn), *Eimeria tenella* (Et), *Theileria annulata* (Ta), *Babesia bovis* (Bb), *Plasmodium falciparum* (Pf), *Plasmodium berghei* (Pb), *Cryptosporidium parvum* (Cp), *Gregarinicae* (G) of the selected genes. (B) Immunofluorescence of intracellular parasites using anti-Ty antibodies (green). An anti-ARO antibody was used to stain the rhoptry organelles (magenta). Counter-staining of DNA with DAPI (blue). Scale bar = 2 μm. (C) Plaque assays with and without rapamycin (Rapa) were conducted to evaluate the overall fitness cost of depleting the selected candidates. The image represents data from 3 biologically independent experiments. Source data are provided as S1 Data. LOPIT, Localization of Organelle Proteins by Isotope Tagging; SP, signal peptide; TM, transmembrane domain.

In the recent years, advancements in genome- and proteome-wide experimental data sets have enhanced our understanding of apicomplexan parasite biology. To uncover novel rhoptry proteins critical in *T*. *gondii*, we integrated these data sets and prioritized genes meeting the following criteria: (1) encoding uncharacterized or hypothetical proteins predicted to localize to rhoptries based on Localization of Organelle Proteins by Isotope Tagging (LOPIT); (2) encoding proteins with predicted transmembrane domains or signal peptides; and (3) showing a negative fitness score from a global CRISPR/Cas9 screen (≤ −2) [24]. According to these criteria, 4 candidate genes were chosen (Fig 1A). Inducible-knockdown parasites were then created by epitope tagging and fusion with the DiCre-mediated conditional U1 gene silencing system at the 3′-untranslated region (UTR) of the gene loci (S1A Fig). This approach uses a parasite line expressing a split Cre recombinase (DiCre) [25]. Treatment with rapamycin (Rapa) triggers Cre dimerization, resulting in excision of the sequence between the LoxP sites and substitution of the exogenous 3′ UTR of the gene of interest with a repeat of 4 U1 recognition sequences, leading to RNA degradation. The CRISPR-Cas9-mediated tagging was conducted at the endogenous locus for all 4 candidates using the 3xTy-U1 cassette to investigate their localization and function. Correct integration, replacement of the 3′ UTR, and rapamycin-induced DiCre-mediated excision were analyzed by genomic PCR (S1A Fig). Indirect immunofluorescence assay (IFA) showed that all selected proteins are localizing to the rhoptries as they partially co-localize with ARO, a marker of the entire rhoptry surface (Fig 1B). Two of them (TGGT1_242820 and TGGT1_276210) are mostly found associated with the bulbous part of the rhoptries while others (TGGT1_305590 and TGGT1_315470) are more apical. Effective reduction of protein expression was observed upon addition of Rapa, as confirmed by western blot analysis, with protein levels significantly decreased after 72 h of treatment (S1B Fig). Plaque assays were conducted to evaluate the collective effect of protein depletion on the parasite lytic cycle. While 3 parasite lines (TGGT1_305590-3Ty-U1, TGGT1_276210-3Ty-U1, and TGGT1_315470-3Ty-U1) exhibited similar lysis plaque formation compared to the parental strain, depletion of TGTT1_242840 resulted in a significant reduction in parasite fitness.

### RDF3 is dually localized, at the apical tip of the rhoptry and associated to the rhoptry bulb

**Fig 2:**
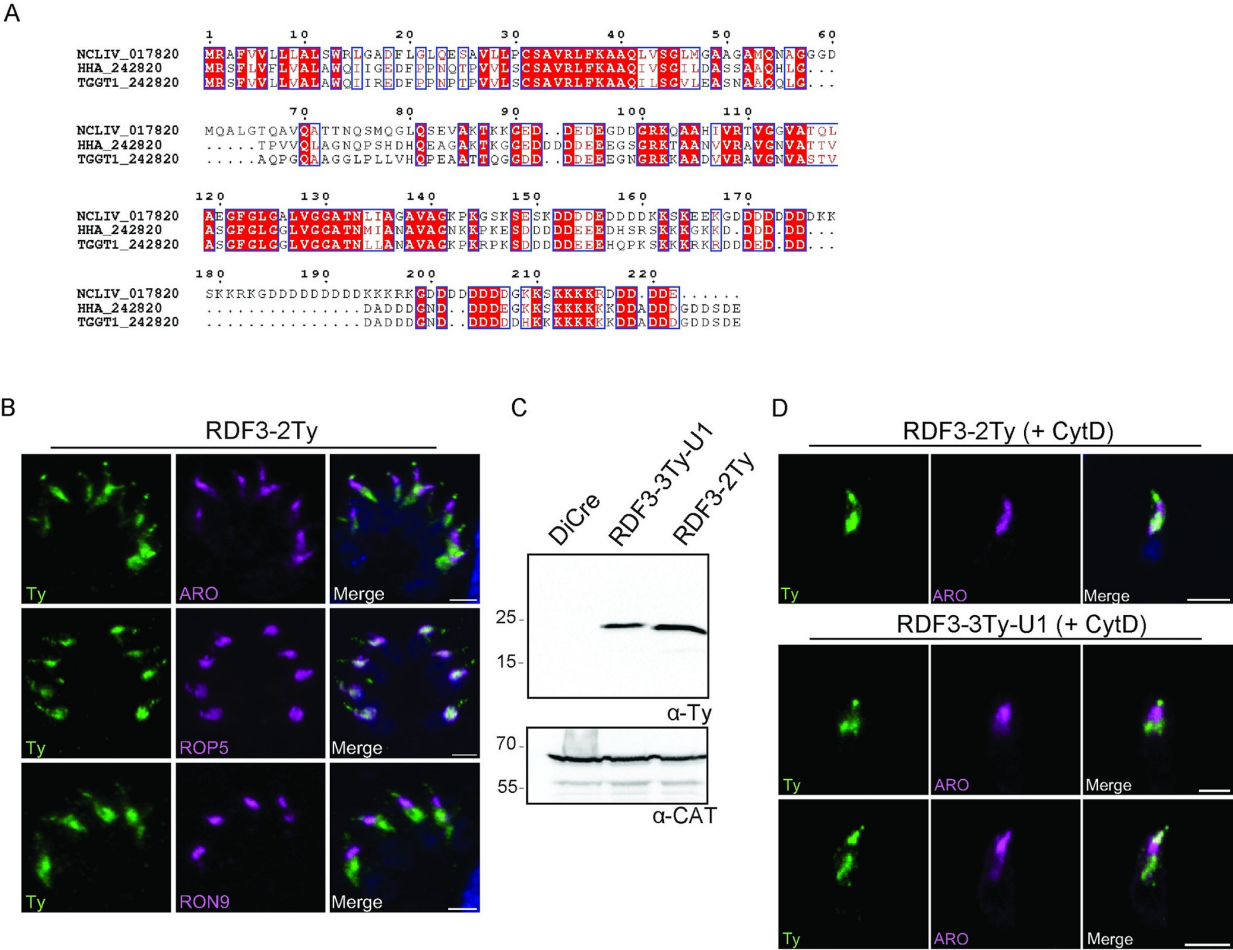
RDF3 localizes to the tip of the rhoptries and associated to the rhoptry bulb. (A) The alignment of TgRDF3 with homologs in *Hammondia Hammondi* (HHA) and *Neospora caninum* (NCLIV). Residues highlighted in red are conserved across the 3 species. (B) Immunofluorescence of intracellular RDF3-2Ty expressing parasites using anti-Ty antibodies (green), anti-ARO (rhoptry membrane), anti-RON9 (rhoptry neck), and anti-ROP5 (rhoptry bulb) antibodies (magenta). Counter-staining of DNA with DAPI (blue). Scale bar = 2 μm. (C) Western blot to assess the level of expression of RDF3 in RDF3-3Ty-U1 and RDF3-2Ty. Catalase (anti-CAT) is used as a loading control. WB representative of 3 biologically independent experiments. (D) Immunofluorescence of extracellular RDF3-2Ty and RDF3-3Ty-U1 parasites in presence of cytochalasin D (+CytD). Counter-staining of DNA with DAPI (blue). Scale bar = 2 μm. Source data are provided as S1 Data. RDF3, rhoptry discharge factor 3.

TGGT1_242820 is a fitness-conferring gene [24], conserved only in a few members of the coccidian subgroup of Apicomplexa, *Hammondia* and *Neospora* species that are closely related to *T*. *gondii* (Fig 1A). The product of TGGT1_242820 is a hypothetical protein of 22 kDa that possesses a predicted signal peptide clustered with the rhoptries by LOPIT [24] (Fig 1A and 1B). This small protein exhibits alternative stretches of acidic and basic amino acid residues but no identifiable domains (Fig 2A). Three regions, C31-Q41, the hydrophobic region G113-A123 and the stretch of charged amino acids at the C-terminus are highly conserved among the putative apicomplexan orthologues (Fig 2A). Based on the functional evidence described below, the gene was named RDF3. CRISPR-Cas9 mediated carboxy-terminal 2Ty-epitope tagging at the endogenous locus (S2A Fig) refined the rhoptry localization of RDF3 (Fig 2B). Intracellular RDF3-2Ty parasites demonstrate a dual localization pattern, with most of the signal colocalizing with ROP5, a marker of the rhoptry bulb. Moreover, an additional RDF3 signal was observed above the RON9 signal, which denotes the neck of the rhoptries (Fig 2B). This dual localization was not observed in intracellular RDF3-3Ty-U1 parasites wherein RDF3 was only visible in the bulb of the rhoptry (Fig 1B). This difference in the RDF3 signal observed between both strains might be explained by the fact that the integration of the LoxP sequences within the 3′ UTR in RDF3-3Ty-U1 parasites leads to a slight decrease in the level of protein expression as observed by western blot (Figs 2C and S2B). This phenomenon has been reported for other proteins [27]. Surprisingly, even though no staining was observed at the rhoptry tip in RDF3-3Ty-U1 intracellular strains, extracellular parasites treated with Cytochalasin D (+ CytD) in presence of host cells, showed an accumulation of the protein at the tip comparable to RDF3-2Ty (Fig 2D). CytD is an inhibitor of actin polymerization, which blocks parasite motility and invasion without impairing rhoptry and microneme secretion [28,29]. This allows us to observe parasites attached to the host cell that have discharged their rhoptries. This result suggests an accumulation of RDF3 at the tip of the rhoptries at the time of invasion likely close to the AV. Moreover, RDF3’s dual localization remains consistent whether the parasite has a retracted (+BIPPO/+CytD) or extruded conoid (S2C Fig).

RDF3 is also detectable in the nascent daughter cells as shown by colocalization with RON9 and IMC1 used to detect the rhoptries/pre-rhoptries and the inner membrane complex (IMC) of daughter cells (S2D Fig). Altogether, RDF3 exhibits a unique dual localization and accumulates to the apical part of the rhoptry during invasion.

### RDF3 is essential for host cell entry by *T*. *gondii* tachyzoites

**Fig 3:**
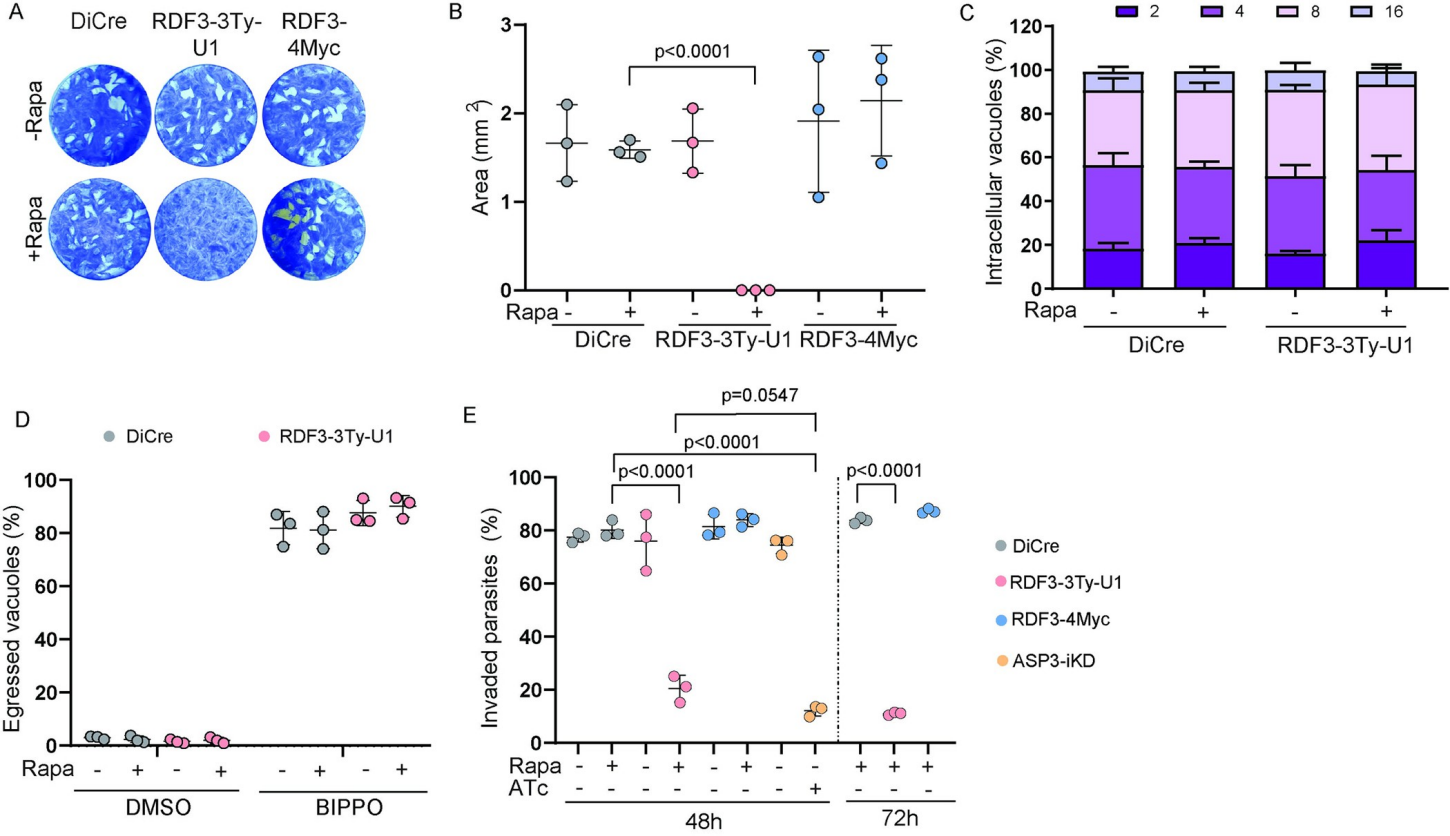
RDF3 is essential for *T*. *gondii* invasion. (A) Plaque assay of different parasite strains (±Rapa) showing that depletion of RDF3 impairs the parasite lytic cycle (no plaque), a phenotype that is fully rescued by complementation with RDF3 wild-type copy (RDF3-4Myc). Image representative of 3 biologically independent experiments. (B) Quantification of plaque assays for DiCre, RDF3-3Ty-U1, and complemented RDF3 strain (±Rapa). (Mean ± SD; *n* = 3 biologically independent experiments.) Statistical significance was assessed by a two-way ANOVA significance with Tukey’s multiple comparison. (C) Intracellular replication assay. Graph representing the number of parasites per vacuole observed at 36 h post-invasion. (Mean ± SD; *n* = 3 biologically independent experiments.) (D) Induced egress assay. Graph representing the percentage of ruptured vacuoles following treatment with the egress inducers BIPPO for DiCre and RDF3-U1-3Ty parasites (±Rapa, 48 h). (Mean ± SD; *n* = 3 biologically independent experiments.) (E) Red/green invasion assay (±Rapa 48 h and 72 h) showing that depletion of RDF3 impairs the invasion of the parasite. ASP3-depleted parasites were used as a negative control. (Mean ± SD; *n* = 3 biologically independent experiments.) A parametric unpaired *t* test was used to assess significance; the two-tailed *p*-values are written on the graphs. Source data are provided as S1 Data. RDF3, rhoptry discharge factor 3.

As previously shown, depletion of RDF3 led to a dramatic loss of parasite fitness when assessed by plaque assay (Fig 1C). Complementation of RDF3-3Ty-U1 was performed by targeting at the *UPRT* locus, a second copy of RDF3 expressed under its own promoter and tagged with 4xMyc tags (RDF3-4Myc) (S3A and S3B Fig). RDF3-4Myc shows the same dual localization as RDF3-2Ty tagged strain (S3C Fig) and fully rescued the phenotype in the presence of rapamycin (Fig 3A and 3B).

To dissect the consequences of RDF3-depletion on parasite fitness, we carried out specific assays that targeted each step of the lytic cycle. The intracellular growth and induced egress from host cell were comparable between the control and RDF3-depleted parasites (Fig 3C and 3D). Conversely, host cell invasion was severely impaired upon 48 h/72 h depletion of RDF3, when assessed by “red-green invasion assay” (Fig 3E) and comparable to the depletion of ASP3, an aspartyl protease previously shown to be essential for *T*. *gondii* invasion and severely impaired in rhoptry discharge [30].

### RDF3 plays a critical role in rhoptry discharge

**Fig 4:**
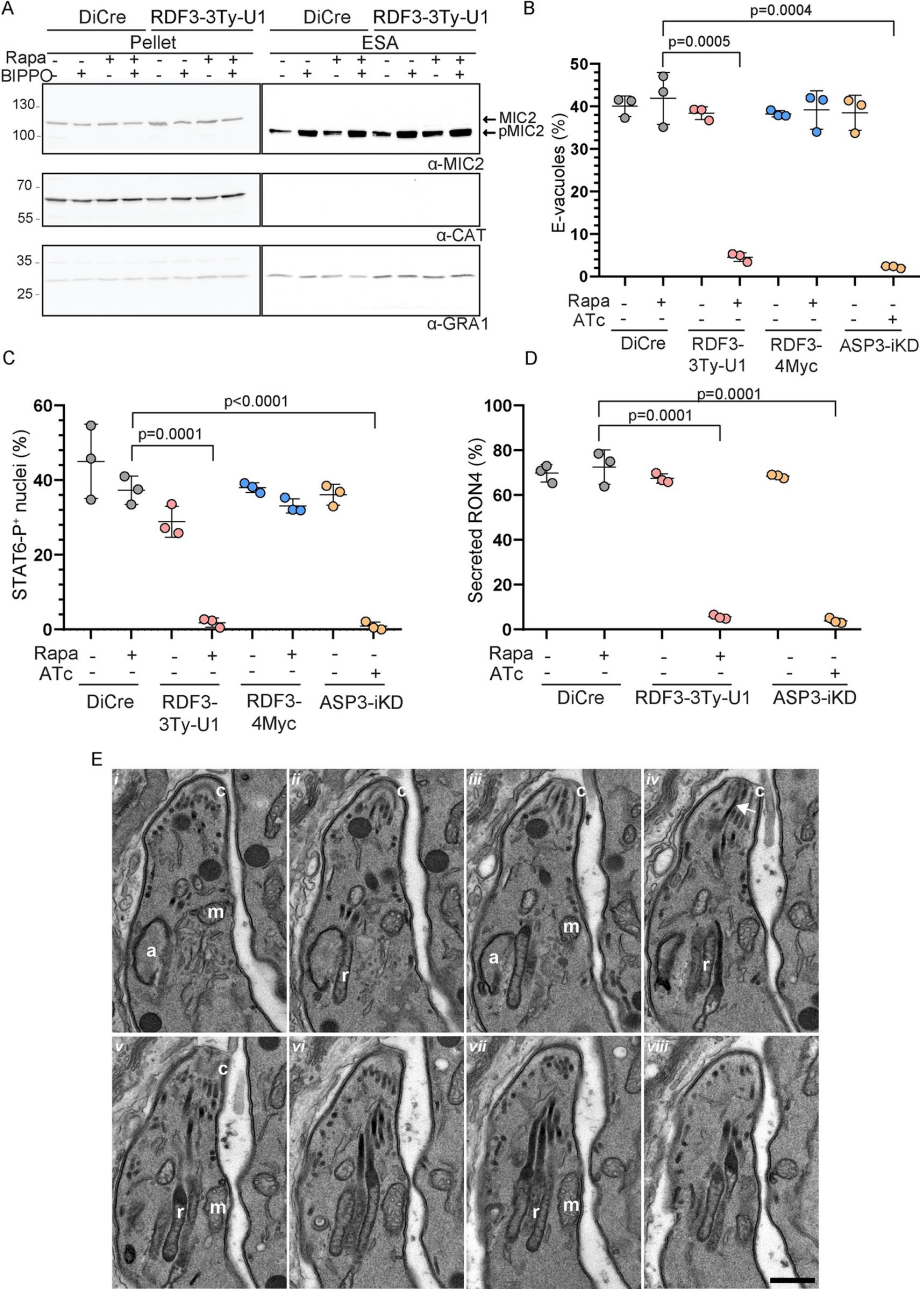
RDF3 is essential for rhoptry discharge. (A) Microneme secretion of extracellular parasites stimulated with BIPPO to assess the release of MIC2 in culture supernatant. ESA excreted–secreted antigens. GRA1 is used as a control for constitutive secretion from dense granules. Samples derived from the same experiment and gels were processed in parallel. Image representative of 3 biologically independent experiments. (B) E-vacuole assays assessing the ability of the parasites to secrete the rhoptry protein ROP1 into the host cell. (Mean ± SD; *n* = 3 biologically independent experiments.) A parametric unpaired *t* test was used to assess significance; the two-tailed *p*-values are written on the graphs. (C) Phospho-STAT6 assays assessing the ability of the parasite to secrete the rhoptry protein ROP16 into the host cell that phosphorylates host STAT6 in the nucleus. (Mean ± SD; *n* = 3 biologically independent experiments.) A parametric unpaired *t* test was used to assess significance; the two-tailed *p*-values are written on the graphs. (D) Quantification of secreted RON4 at the tip of CytD-treated parasites. (Mean ± SD; *n* = 3 biologically independent experiments.) A parametric unpaired *t* test was used to assess significance; the two-tailed *p*-values are written on the graphs. (E) ssTEM analysis confirmed the normal morphology and positioning of rhoptries in absence of RDF3 in intracellular parasites. Shown is gallery of 8 consecutive sections (i–viii) at 70 nm thickness through the one tachyzoite highlighting the normal rhoptries (r) morphology and bundled positioning at the apical tip of the parasite, together with one rhoptry neck docked (arrow in panel iv) inside the conoid (c). Shown also are the apicoplast (a) and mitochondrion (m). Scale bar: 0.5 μm. Source data are provided as S1 Data. RDF3, rhoptry discharge factor 3; ssTEM, serial section transmission electron microscopy.

The concerted action of microneme secretion and rhoptry discharge is essential for parasite invasion. We therefore examined whether these organelles were properly secreted in RDF3-depleted parasites. To assess microneme secretion integrity in RDF3-depleted parasites, we induced secretion with BIPPO and quantified processed MIC2, a secreted microneme protein, in the supernatant via western blot. A comparable amount of secreted MIC2 was found in the supernatant of the control strain (DiCre) and RDF3-depleted parasites, indicating no defect in microneme secretion (Fig 4A). We then examined the ability of the parasite to discharge rhoptry content in the absence of RDF3 by 3 different methods assessing the discharge of membranous materials and lipids (e-vacuoles assay), the injection of ROP16 (STAT6-phosphorylation assay), and the secretion of RON4 at the contact point between the parasite and the host cell (RON4-dot). RDF3-depleted parasites exhibited a significant impairment in rhoptry discharge, as evidenced by the 3 assays (Fig 4B–4D), which explains the observed invasion blockade. Unlike ASP3 depletion, which results in aberrant rhoptry morphology and positioning, rhoptries in RDF3-depleted parasites maintained proper morphology and positioning at the apical tip of intracellular parasites, as observed via serial section transmission electron microscopy (ssTEM) (Fig 4E). Moreover, rhoptry proteins are properly expressed and targeted to the organelles in absence of RDF3, as showed with RON9 and ROP5 that exhibit a normal localization, expression and processing assessed by IFA (S4A Fig) and western blot (S4B Fig).

### RDF3 dynamically delocalizes upon host cell entry

**Fig 5:**
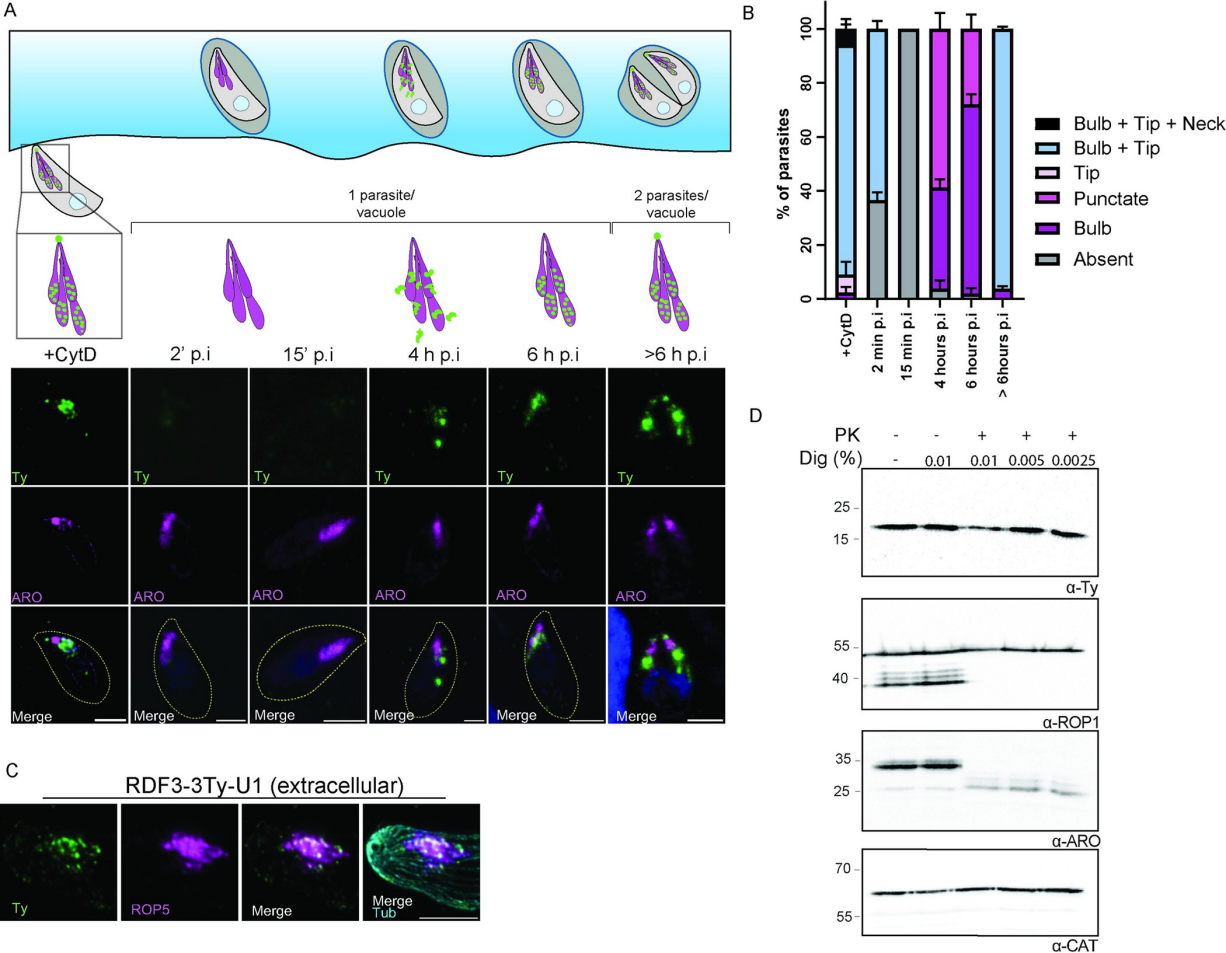
RDF3 is a highly dynamic protein. (A) Immunofluorescence of RDF3-2Ty parasites fixed at different time points post-invasion. Anti-Ty antibody (green) is used to stain RDF3. Anti-ARO antibody is staining the rhoptry organelle (magenta). Counter-staining of DNA with DAPI (blue). Scale bar = 2 μm. (B) Quantification of RDF3 staining at different time points post-invasion (*n* = 3 biologically independent experiments). (C) U-ExM pictures for localization of RDF3-3Ty-U1 in extracellular parasites. Expansion factor = 4. Scale bar = 1 μm. (D) Proteinase K protection assay demonstrating that RDF3-2Ty is protected upon permeabilization and protease treatment. ARO and ROP1 were used as controls for the surface and lumen of rhoptries, respectively. Dig = digitonin, PK = proteinase K. Source data are provided as S1 Data. RDF3, rhoptry discharge factor 3.

Rhoptry secretion relies on an arsenal of proteins located at the RSA at the tip of the parasite [3,4,15]. Some components of the apparatus might be perennial while others might show a dynamic localization when rhoptry discharge occurs. Therefore, we sought to dissect the dynamics of RDF3 localization by performing a time course infection using RDF3-2Ty expressing parasites. The invasion was synchronized by preincubation on ice and then started by incubating the infected culture at 37°C. Samples were fixed at different time points and a drastic change in RDF3 localization was observed over the course of invasion (Fig 5A). The percentage of intracellular parasites at each time point was quantified (S5A Fig) together with the localization of RDF3. In extracellular parasites treated with CytD (block of invasion), RDF3 is found at the bulb and at the tip in 90% of parasites. RDF3 signal is absent in 40% of parasites at 2 min post-invasion, coinciding with only 50% of parasites being internalized (Fig 5A and 5B). This likely accounts for the observed signal retention in the bulb and tip in 60% of parasites by IFA and the detectable signal by western blot (Figs 5B, S5A and S5B). In contrast, RDF3 was undetectable in internalized parasites at 15 min to 1 h post-invasion by IFA and WB (Figs 5A, 5B and S5B). After 4 h, the signal of RDF3 reappeared as a punctate staining observed around the rhoptry in 60% of the parasites, whereas in almost 35% the staining was observed at the bulb (Fig 5A and 5B). At that time, the parasite has not yet replicated and therefore the punctuated RDF3 staining is likely distinct from de novo rhoptry organelle biogenesis seen during daughter cell formation. Six hours post-invasion, a staining around the bulb is observed in almost 70% of the parasites with a punctate signal still observed in around 25% of the parasites (Fig 5A and 5B). Finally, when the parasite starts to replicate (>6 h post-invasion), a staining is observed at the tip and at the rhoptry bulb (Fig 5A and 5B). To refine the localization of RDF3 at a higher resolution, we employed ultrastructure Expansion Microscopy (U-ExM) (36). U-ExM analysis of extracellular RDF3-3Ty-U1 parasites revealed that RDF3 indeed localizes to the rhoptry bulb. However, its signal appears punctate and more dispersed compared to ROP5 staining (Fig 5C). The absence of RDF3 signal at the rhoptry tip using this technique raises questions about its localization. These findings, coupled with RDF3’s expression profile differing from luminal rhoptry proteins, prompt further investigation into whether RDF3 resides inside the rhoptry lumen or on the organelle surface (S5C Fig). To elucidate RDF3’s topology, we performed proteinase K protection assay (Fig 5D). We subjected parasites to digitonin and proteinase K treatment, followed by western blot. Under these conditions RDF3, like the luminal rhoptry protein ROP1, was not proteolytically degraded, suggesting that it is not exposed to the cytosol. In contrast the rhoptry surface protein ARO was degraded (Fig 5D). Also, immuno-electron microscopy (EM) was performed on RDF3-2Ty strains, but the results did not yield any additional data to resolve RDF3 localization (S5D Fig). The gold particles were found scattered randomly throughout the cytoplasm, nucleus, and occasionally on the bulb of rhoptries.

Additionally, tagging the C-terminus of RDF3 with a “mini auxin-induced degron” (mAID) cassette, confirmed that RDF3 is in the lumen of the secretory pathway and its C-terminus is not exposed to the cytosol as addition of indole-3-acetic acid (IAA) does not lead to its proteasome-mediated degradation (S5E Fig). IFA analysis and plaque assays validate the proper targeting and functionality of RDF3-mAID-3Ty (S5F–S5H Fig). RDF3-3Ty-U1 is soluble in PBS indicating that the protein is not associated with membrane and that the N-terminal putative signal peptide is cleaved (S5I Fig).

### RDF3 localization close to the AV is independent of rhoptry biogenesis and positioning

**Fig 6:**
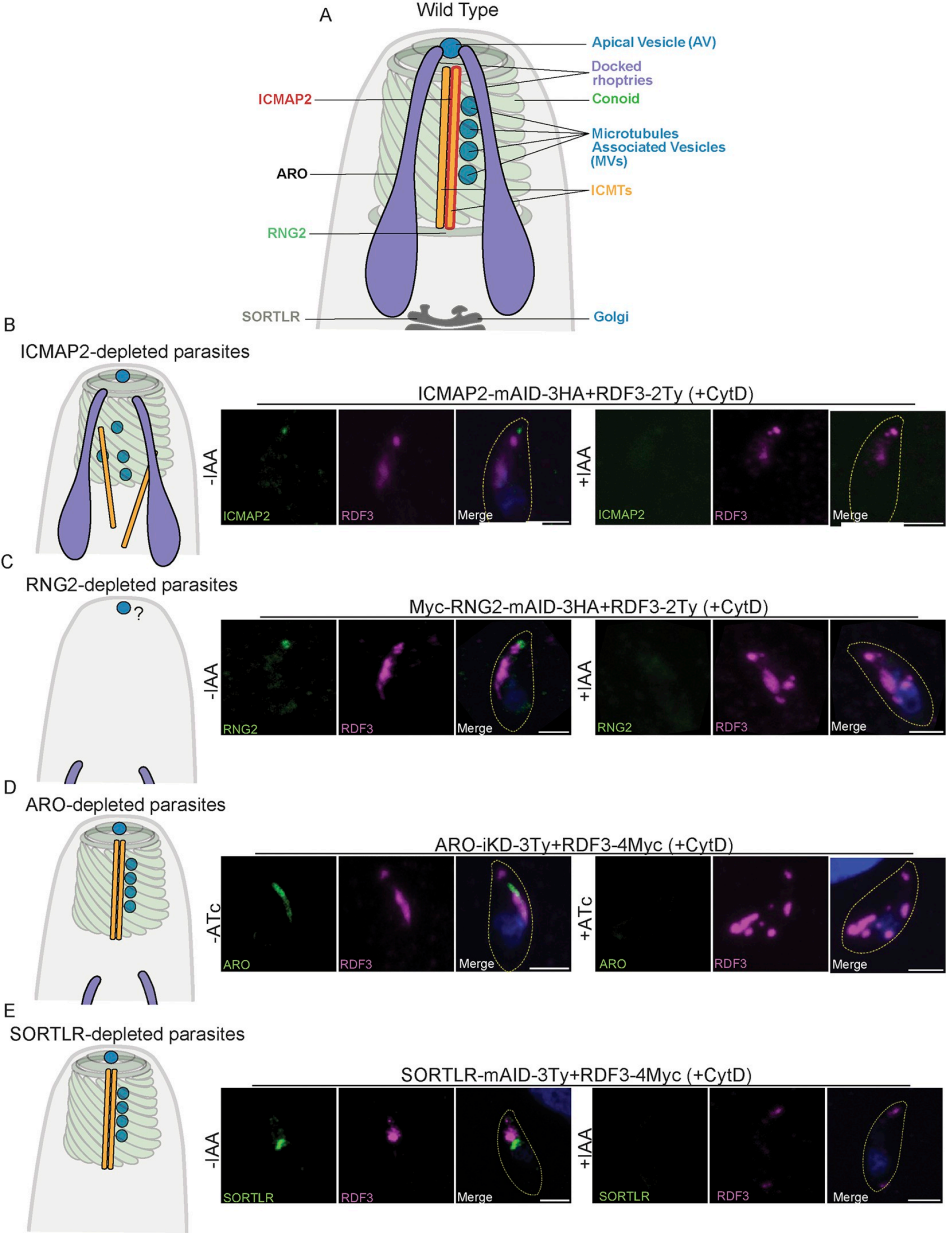
The apical localization of RDF3 is independent of rhoptries. (A) Schematic representation of the apical complex of *T*. *gondii* highlighting the main structures and molecular players down-regulated in this study. (B) Immunofluorescence to assess RDF3 localization in ICMAP2-depleted parasites in extracellular parasites in presence of cytochalasin D (+CytD). Counter-staining of DNA with DAPI (blue). Scale bar = 2 μm. (C) Immunofluorescence to assess RDF3 localization in RNG2-depleted parasites in extracellular parasites in presence of cytochalasin D (+CytD). Counter-staining of DNA with DAPI (blue). Scale bar = 2 μm. (D) Immunofluorescence to assess RDF3 localization in absence of ARO in extracellular parasites in presence of cytochalasin D (+CytD). Counter-staining of DNA with DAPI (blue). Scale bar = 2 μm. (E) Immunofluorescence to assess RDF3 localization in absence of SORTLR in extracellular parasites in presence of cytochalasin D (+CytD). Counter-staining of DNA with DAPI (blue). Scale bar = 2 μm. Source data are provided as S1 Data. ICMAP2, intraconoidal microtubules associated protein 2; RDF3, rhoptry discharge factor 3.

To dissect RDF3 localization relative to the perturbation of components of conoid, we tagged the protein in 2 parasite lines that conditionally regulate the expression of genes orchestrating this structure (Fig 6A). First, RDF3 was tagged in the intraconoidal microtubules associated protein 2 (ICMAP2)-mAID-3HA strain. Depletion of ICMAP2 leads to the dissociation of the 2 ICMTs, their detachment from the conoid and the dispersion of the MVs and rhoptries [17]. Under this condition, RDF3 localization is not affected in intracellular parasites (S6A Fig) or in extracellular parasites treated with CytD (Figs 6B and S6B). Subsequently, RDF3 was tagged in the Myc-RNG2-mAID-3H strain. RNG2 is known to have a dual localization at the base of the conoid and the apical polar ring. Depletion of RNG2 results in the detachment of the conoid, ICMTs, MVs, and leads to a disorganization of the rhoptries [31]. In the absence of a molecular marker for the AV, the impact of RNG2 depletion on its fate remains unclear. Nevertheless, under this condition, RDF3 maintains normal localization at the tip, whereas the staining of the rhoptry bulb becomes disorganized, indicating that the bulb-associated localization of RDF3 follows the disorganization of the rhoptries (Figs 6C, S6C and S6D).

Next, we introduced a second copy of RDF3 under its own promoter (RDF3-4Myc) in the inducible knock-down mutants of ARO and SORTLR (Fig 6D and 6E). In the absence of ARO, rhoptries become detached from the apical complex and disperse in the cytosol (Figs 6D, S6E and S6F) [7]. In ARO-depleted parasites treated with CytD, RDF3 dispersed in the cytosol, but its apical localization remained unaffected. Similarly, in SORTLR-depleted parasites treated with CytD, the localization of RDF3 at the rhoptry bulb was lost, while its localization at the tip remained unchanged, albeit with decreased intensity (Figs 6E and S6H).

Overall, RDF3’s localization at the rhoptry bulb appears closely linked to the organelles, while its positioning at the apex persists even in the absence of the conoid and rhoptries. This suggests that RDF3 at the parasite’s apex may be linked to a structure unaffected by RNG2 depletion, potentially the AV.

### RDF3, RASP2, and CSCHAP are independently positioned at the tip of the parasite

To explore potential RDF3-interacting partners, we initially employed proximity labeling by introducing a second copy of RDF3 fused with the biotin-labeling protein, MiniTurbo [32] at the C-terminus. However, IFA indicated that the addition of this C-terminal tag disrupted the proper localization of RDF3 (S7A Fig). Consequently, we shifted our focus to investigating RDF3’s localization relative to proteins known to localize at the apex of the parasite. We also monitored how RDF3 localization was affected when these proteins were depleted. Nd6, RASP2, and CSCHAP are recognized to localize near the AV and play crucial roles in rhoptry discharge, albeit their precise functions remain unclear.

**Fig 7:**
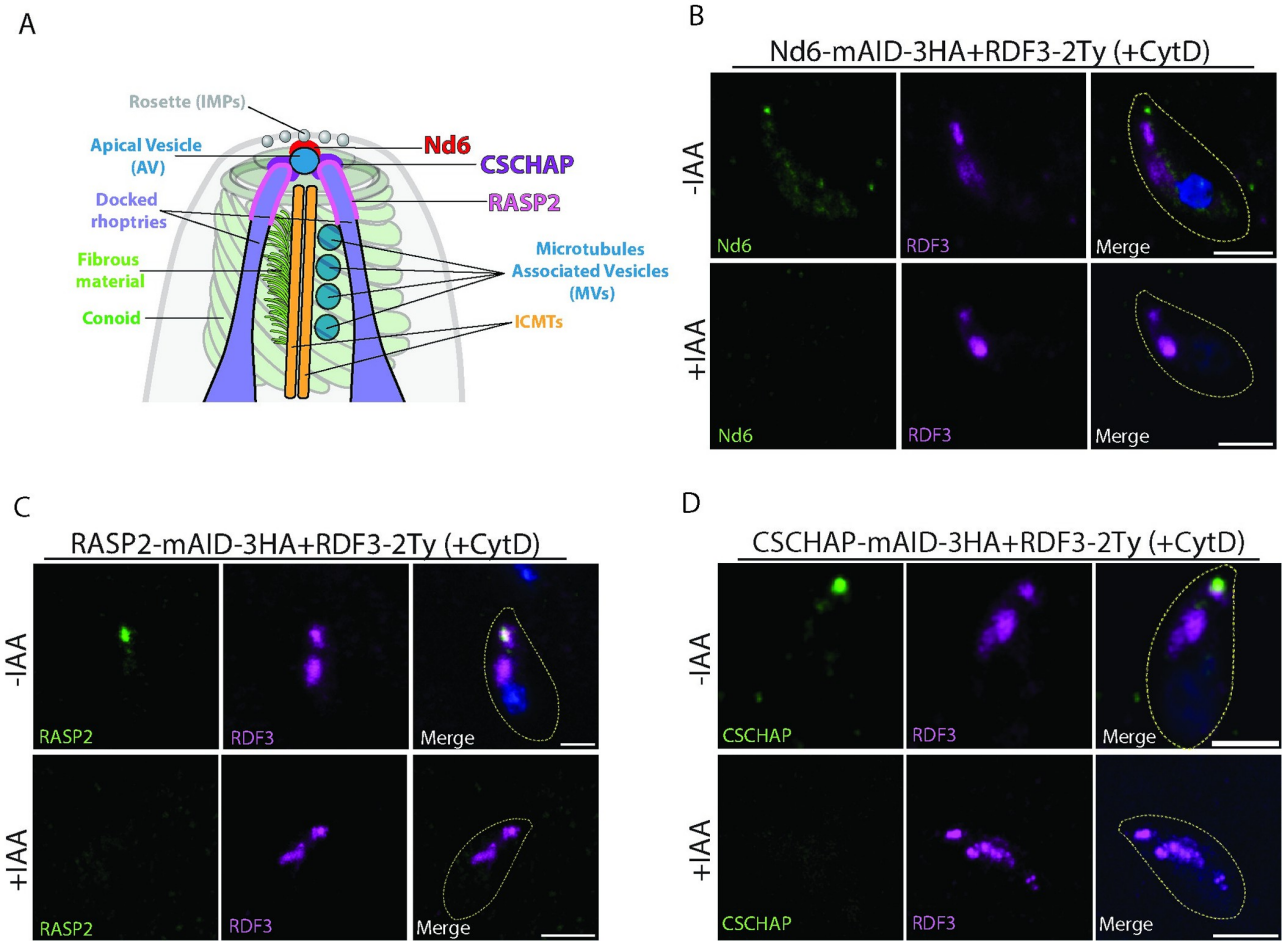
RDF3 partially colocalizes with RASP2 and CSCHAP at the parasite tip. (A) Schematic representation of the main molecular players required for rhoptry secretion and localizing close to the AV. (B) Immunofluorescence to assess RDF3 localization with Nd6 in extracellular parasites in presence of cytochalasin D (+CytD). (C) Immunofluorescence to assess RDF3 localization with RASP2 in extracellular parasites in presence of cytochalasin D (+CytD). Counter-staining of DNA with DAPI (blue). Scale bar = 2 μm. (D) Immunofluorescence to assess RDF3 localization with CSCHAP in extracellular parasites in presence of cytochalasin D (+CytD). Source data are provided as S1 Data. AV, apical vesicle; RASP2, rhoptry apical surface protein 2; RDF3, rhoptry discharge factor 3.

First, to determine if RDF3 localization is dependent on these proteins, we endogenously tagged RDF3 in parasites strains where proteins are C-terminally fused to mAID-3HA cassette at the endogenous loci. The signal of RDF3 at the apex did not colocalize with Nd6, but it partially colocalized with RASP2 and CSCHAP signals in extracellular (Fig 7B–7D, upper lanes) as well as in intracellular parasites (S7B Fig). Depletion of Nd6, RASP2, or CSCHAP did not affect the localization of RDF3 neither at the rhoptry bulb nor at the apex (Fig 7B–7D, lower lanes). Reciprocally, RASP2 and CSCHAP were epitope tagged in the RDF3 inducible-knockdown parasites and confirmed that their apical localizations were not affected in the absence of RDF3 (S7C Fig). However, analysis of CSCHAP and RASP2 dynamics during invasion revealed that unlike RDF3, the signals of the 2 proteins persist even after parasite internalization (S7D Fig). Collectively, these findings suggest that while RDF3, RASP2, and CSCHAP exhibit some colocalization and play roles in rhoptry secretion, they do not rely on each other for targeting to the apical tip of the parasite.

### Cryo-electron tomography reveals a contribution of RDF3 in MV biogenesis

**Fig 8:**
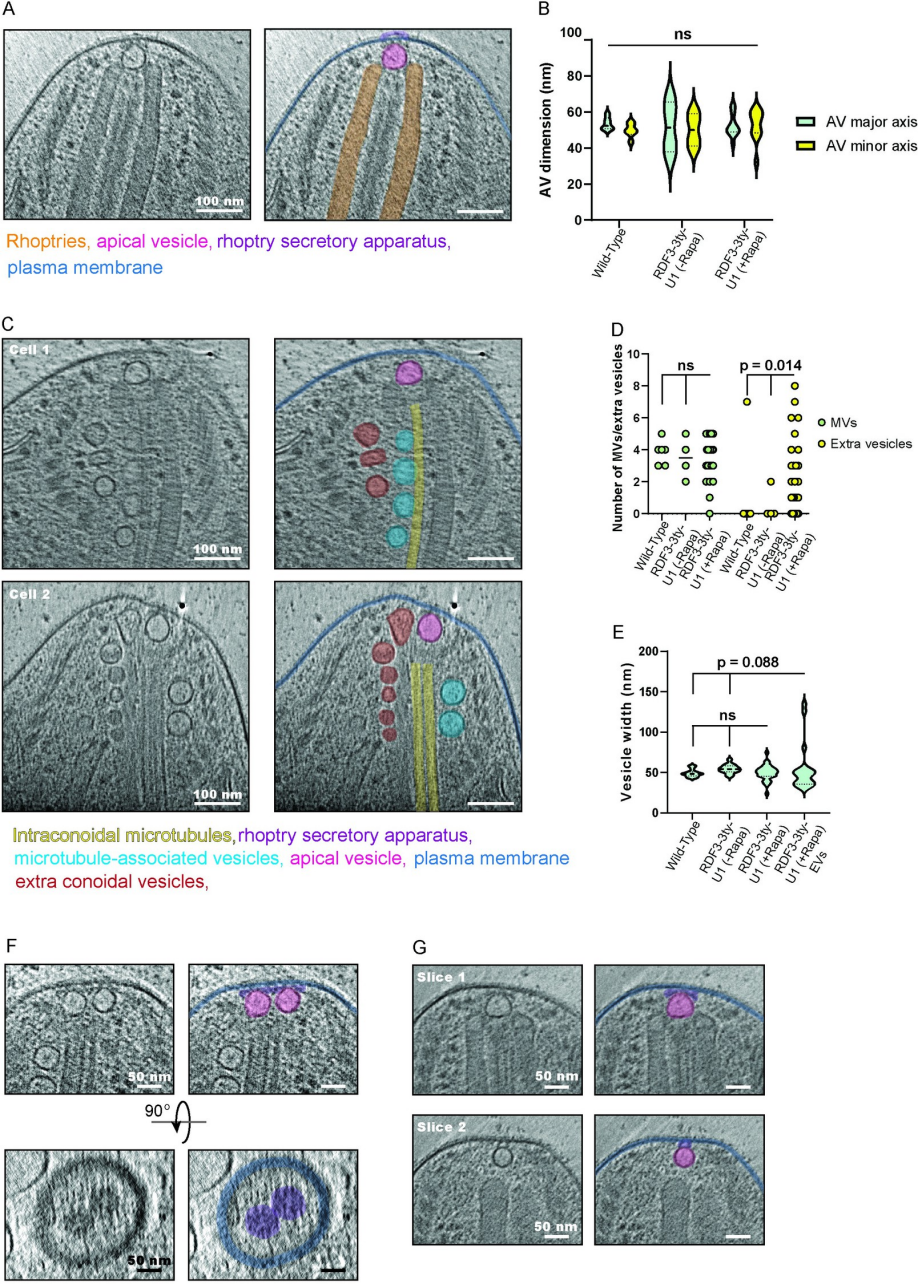
Cryo-electron tomography of the apical end of RDF3 knockdown *T*. *gondii*. (A) 2D slice through a tomogram of an RDF3 knockdown *T*. *gondii*, highlighting the components of the rhoptry secretion system. (B) Violin plot of the AV dimensions in wild-type, RDF3 control, and RDF3 knockdown parasites. A two-sided *T* test was performed to determine statistical significance between the knockdown and the control and wild-type together. *N* = 6 wild-type, 4 RDF3 control, and 10 RDF3 knockdown parasites. (C) 2D slice through 2 tomograms (top and bottom) of RDF3 knockdown parasites, highlighting extra conoidal vesicles forming an array, comparable to the MVs. (D) Plot of the number of MVs (left half) and extra conoidal vesicles (right half) in wild-type, RDF3 control, and RDF3 knockdown parasites. A two-sided Mann–Whitney U test was performed to determine statistical significance between the RDF3 knockdown and the control and wild-type together. *N* = 6 wild-type, 4 RDF3 control, and 20 RDF3 knockdown parasites. (E) Violin plot of the MV widths in wild-type, RDF3 control, and RDF3 knockdown parasites, and extra conoidal vesical widths in the knockdown parasites. A two-sided *T* test was performed to determine statistical significance between MVs or extra vesicles from the knockdown and MVs from the control and wild-type together. *N* = 6 wild-type, 4 RDF3 control, and 10 RDF3 knockdown parasites. (F) Side view (top) and top-down view (bottom) through a tomogram of an RDF3 knockdown parasite that has 2 AVs and 2 RSAs. (G) Different slices through one tomogram of an RDF3 knockdown parasite displaying a normal AV and RSA (top) and another AV-like vesicle with a partially formed RSA (bottom). Panels A, E–G are without (right) and with (left) color overlay. Scale bars are 100 nm in panels A and E, and 50 nm in panels F and G. Statistical significance was written as “ns” if *p* > 0.1. Source data are provided as S1 Data. AV, apical vesicle; MV, microtubule-associated vesicle; RDF3, rhoptry discharge factor 3; RSA, rhoptry secretion apparatus.

Given the apical localization of RDF3 and its crucial role in rhoptry secretion, we hypothesized that knockdown of RDF3 would result in a significant structural defect of the rhoptry secretion machinery. Upon imaging of RDF3 knockdown extracellular parasites, surprisingly there were no obvious structural changes around the rhoptries. We observed normal rhoptry docking to the AV and a similarly shaped AV compared to both the wild-type parasites and control parasites without the induction of RDF3 knockdown (Figs 8A, 8B and S8A). Examination of the vesicles associated with the ICMTs showed that RDF3 knockdown parasites had an abundant number of vesicles in the vicinity of the ICMTs (Fig 8C). Some vesicles are clearly associated with the ICMTs and were identified as MVs (Fig 8C, blue) while others were found more distant and were named extra vesicles (EVs) (Fig 8C, brown). Quantification of the MVs number and size showed no significant differences between control and RDF3-depleted parasites (Fig 8D). When the same analysis was performed on EVs, their number varied significantly between control and RDF3-depleted parasites and their size appeared more variable even if not significantly different from the control (Figs 8D, 8E, S8B and S1–S4 Movies). Moreover, 4 out of 45 knockdown parasites displayed 2 AVs and 2 RSAs (Fig 8F). The phenomenon of 2 AVs has also been noted in the ICMAP2-iKD mutant [17], which resulted in disrupted MVs arrangement. The presence of a second AV might arise from premature anchoring of an MV to the cell apex to replenish the AV [17]. We additionally observed one parasite with a tentative second AV associated to an incomplete RSA (Fig 8G), potentially in the process of RSA biogenesis. Interestingly, there is still a chain of MVs near the ICMTs, but they display variable sizes compared to the control parasites (Fig 8E). Taken together, RDF3 appears to play a role in the proper trafficking, number, and morphology of the MVs.

### Functional dissection of the conserved amino acids sequences of RDF3

**Fig 9:**
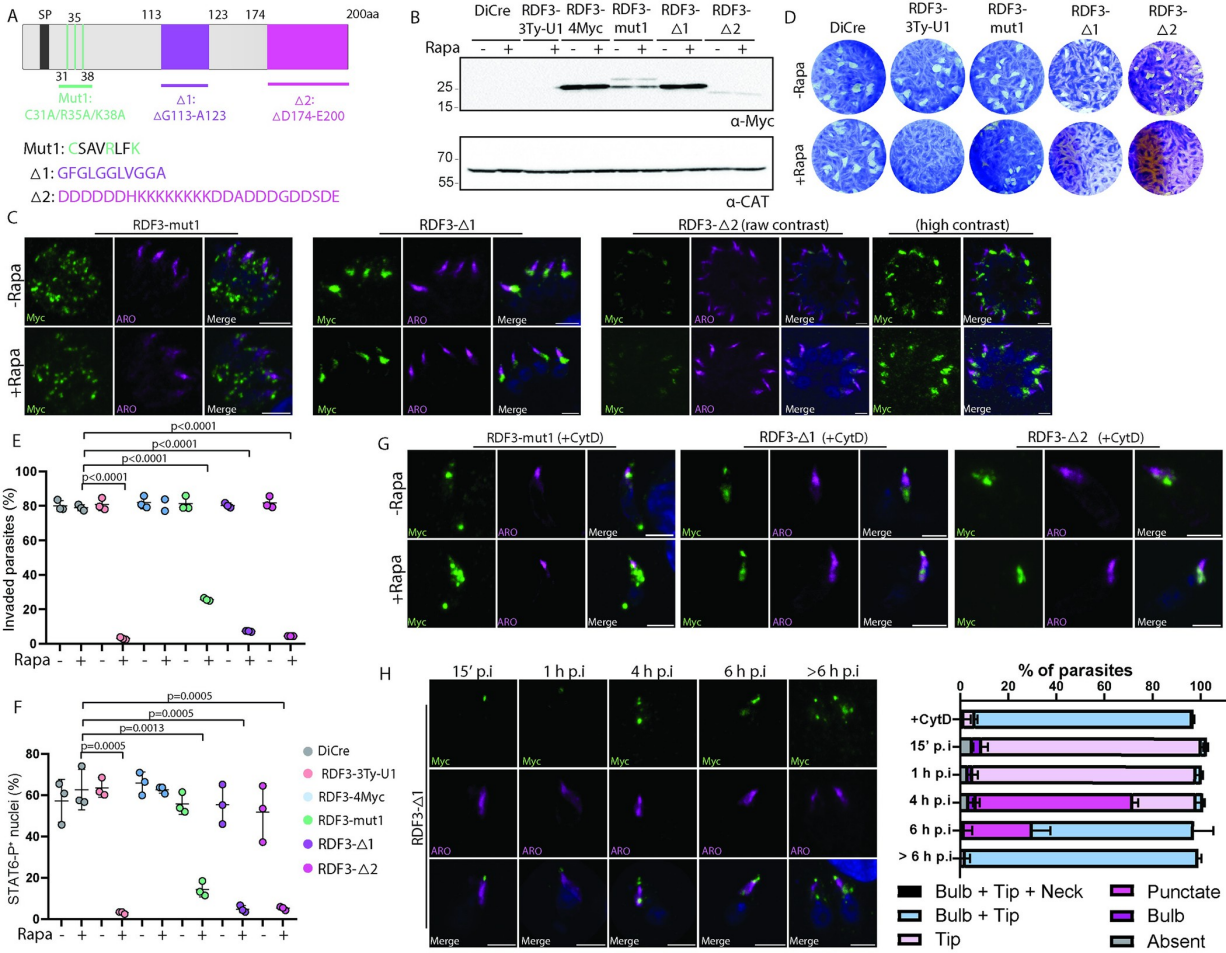
Conserved regions in RDF3 are important for the protein trafficking and function. (A) Schematic representation of the mutants. Mut1 (green). Amino acids highlighted in green were mutated to alanine. Δ1 (purple); 11 amino acids highlighted in purple were deleted. Δ2 (pink); 20 amino acids highlighted in pink were deleted. (B) Western blot using anti-Myc antibodies showing the expression of the complemented mutated strains compared to the parental line DiCre and RDF3-depleted parasites. Catalase (anti-CAT) is used as a loading control. (C) Immunofluorescence of the 3 intracellular mutant parasites using anti-Myc antibody (green) and anti-ARO antibody (magenta). Scale bar = 2 μm. (D) A representative image of a plaque assay of different parasite strains (±Rapa) showing that complementation using RDF3-Δ1 and RDF3-Δ2 do not rescue the parasite lytic cycle (no plaque), whereas small plaques were observed for RDF3-mut1. (E) Red/green invasion assay (±Rapa) showing that complementation with RDF3 mutants does not rescue the invasion of the parasite. (Mean ± SD; *n* = 3 biologically independent experiments.) A parametric unpaired *t* test was used to assess significance; the two-tailed *p*-values are written on the graphs. (F) Phospho-STAT6 assays assessing the ability of the parasite to secrete the rhoptry protein ROP16 into the host cell that phosphorylates host STAT6 in the nucleus. (Mean ± SD; *n* = 3 biologically independent experiments.) A parametric unpaired *t* test was used to assess significance; the two-tailed *p*-values are written on the graphs. (G) Immunofluorescence of the 3 extracellular mutant parasites (±Rapa) in the presence of cytochalasin D (+CytD). Anti-Myc antibody (green) was used to visualize RDF3 protein and anti-ARO antibody (magenta). Scale bar = 2 μm. (H) Immunofluorescence of RDF3-Δ1 parasites fixed at different time points post-invasion. Anti-Ty antibody (green) is used to stain RDF3. Anti-ARO antibody is staining the rhoptry organelle. Counter-staining of DNA with DAPI (blue). Scale bar = 2 μm. Source data are provided as S1 Data. RDF3, rhoptry discharge factor 3.

RDF3 exhibits no recognizable domain but shows regions of sequence conservation among the apicomplexan homologues (Fig 2A). We reasoned that if conserved, those regions might be functionally relevant. We generated parasite strains expressing 3 different mutated forms of RDF3 as extra copies in RDF3-3Ty-U1 parasites to evaluate their potential to functionally complement RDF3 depletion (Figs 9A and S9A). RDF3-mut1 corresponds to C31, R35, and K38 mutations into alanine residues (Fig 9A, green residues). In RDF3-Δ1, 11 amino acids in the conserved hydrophobic region G113-A123 were deleted (Fig 9A, purple residues), whereas RDF3-Δ2 corresponds to a deletion in the stretch of 27 conserved charged residues in the C-terminal end D174-E200 (Fig 9A, pink residues). One copy of each of these mutated RDF3 cDNAs was individually integrated in the *UPRT* locus and the resulting mutants were confirmed by genomic PCR analysis (S9A Fig). The 3 RDF3 mutants migrated to the expected molecular size (Figs 9B and S9B). We observed an additional faint band migrating a few kDa above RDF3-mut1, and RDF3-Δ2 was detectable only at a very low level (Fig 9B). RDF3-mut1 showed a punctate staining throughout the parasite, more abundant at the basal pole (Fig 9C) while RDF3-Δ1 displayed a normal localization at the bulb and on the rhoptry tip. RDF3-Δ2 lost the staining at the rhoptry tip and has a faint staining in the rhoptry bulb (Fig 9C) probably due to its low expression level as observed by western blot (Fig 9B). The 3 mutants showed the same solubility as RDF3-3Ty-U1 (S9C Fig). Plaque assay revealed that RDF3-mut1 strain was able to form small plaques, partially rescuing the phenotype, whereas RDF3-Δ1 and RDF3-Δ2 formed no plaques (Figs 9D and S9D). The 3 complemented strains showed unchanged intracellular growth compared to the DiCre control and RDF3-depleted parasites (S9E Fig). Host cell invasion was severely impaired upon depletion of the endogenous RDF3, in RDF3-Δ1 and RDF3-Δ2 (Fig 9E). Intriguingly, even though RDF3-mut1 trafficking was perturbed, the invasion defect of this mutant was partially rescued (Fig 9E). RDF3-mut1 parasites showed a slight rescue of rhoptry secretion compared to RDF3-depleted parasites (Fig 9F), in line with the results observed in plaque assay and invasion (Figs 9D and 9E). A severe defect in rhoptry discharge, as measured by STAT6 phosphorylation, was observed in RDF3-Δ1 and RDF3-Δ2 strains, comparable to RDF3-depleted parasites (Fig 9F). In extracellular parasites treated with CytD, RDF3-mut1 accumulated at both parasite apical and basal poles and no punctate staining in the parasite cytoplasm was observed (Fig 9G). Signal for RDF3-Δ1 was similar to WT, whereas RDF3-Δ2 lacked the accumulation at the rhoptry tip (Fig 9G). Since RDF3-Δ1 displayed a normal localization despite the severe defect in invasion and rhoptry discharge, we investigated its dynamic after invasion. Results for the bulb localization between 15 min to >6 h post-invasion were comparable to WT (Figs 9H and 5A). However, the apical localization in RDF3-Δ1 did not disappear after invasion, compared to RDF3-2Ty (Figs 9H and 5A).

Collectively, these results suggest that the N-terminal conserved residues are involved in the correct trafficking of RDF3, while the hydrophobic region plays a crucial functional role in apex localization of the protein, and the C-terminal acidic stretch contributes to protein stability.

## Discussion

Rhoptries represent unique organelles essential for apicomplexan parasites such as *T*. *gondii*, assisting establishment of infection and subverting host cellular functions. The process of rhoptry discharge, crucial for successful invasion, involves intricate temporal and spatial coordination. It requires the secretion of their contents into the host cell while navigating through multiple physical barriers, including the rhoptry membrane, parasite plasma membrane, and host cell plasma membrane. This complex event involves a conserved macromolecular machine, the RSA, which forms a rosette at the parasite apex and docks an AV [9,10,33]. In the coccidian subgroup where there are more than 2 rhoptries, the secretion machinery expands to include 2 ICMTs and a series of up to 6 MVs. This expansion is believed to facilitate iterative rhoptry discharge [9,13]. The rosette, found in ciliates as well, facilitates the fusion of the rhoptry membrane with the parasite plasma membrane and is crucial for the exocytosis of secretory organelles. In contrast, some molecular players of rhoptry secretion such as CSCHAP, RASP2, RDF1, and RDF2 are unique to Apicomplexa and might be tailored to act in the fusion event with the host plasma membrane leading to the delivery of rhoptry contents into the host cytosol [8,21,23]. Moreover, the AV is also unique to Apicomplexa and likely instrumental for this last step, which is mechanistically and molecularly not understood to date. Importantly, the composition of the AV and how the rhoptries are docked to it remain unknown.

Here, we characterized RDF3, a small protein conserved in a subset of apicomplexans possessing multiple rhoptries. Unlike the broadly conserved ARO or ASP3, whose depletion results in mispositioning or morphological alteration of the rhoptries, respectively [7,30,34], depletion of RDF3 blocks rhoptry secretion without affecting organelle positioning or morphology. Although little is known about what triggers rhoptry discharge, it is tightly linked to microneme secretion [21,29] and importantly microneme secretion occurs normally in RDF3-depleted parasites.

RDF3 is a highly charged protein with multiple stretches of acidic and basic residues that may play a role in its interaction with other proteins or membranes, facilitating rhoptry discharge at the time of invasion. Though RDF3 has no homolog in *Plasmodium* spp., the *Plasmodium* Apical Sushi protein (PfASP), also known as PfRON1, contains similar repetitions of negatively charged residues at the C-terminal domain [35]. However, this protein is not fully characterized, and its precise role is not known.

RDF3 shows a dual localization at the rhoptry bulb and close to the AV, not yet reported for any rhoptry protein. The detection of RDF3 at the rhoptry tip appears to be dependent on its level of expression since the introduction of LoxP sequences at the 3′ UTR of the gene lowers RDF3 levels and hampers its detection at the tip in intracellular parasites. Additionally, in intracellular parasites, the signal of RDF3 close to the AV is very faint compared to extracellular parasites. This implies that the protein accumulates at the rhoptry tip in extracellular parasites ready to invade and might be triggered by the attachment of the parasite to a host cell. RDF3 is fully soluble in PBS, suggesting that the N-terminal predicted signal peptide is cleaved. RDF3 seems to be enclosed in the lumen of the rhoptry organelles or vesicles as it is largely protected from degradation either by proteolysis under selective permeabilization conditions or by auxin-mediated proteasomal degradation. However, determining if this applies to both localizations of the protein is challenging due to technical limitations. Interestingly, RDF3 protein becomes undetectable, upon internalization of the parasite (2 min post-invasion). It is unlikely that the protein is secreted into the host cell as parasites treated with CytD retain RDF3 signal. Thus, we hypothesize that RDF3 is likely subjected to degradation process following completion of invasion. The investigation of the AV and MVs as key players in rhoptry secretion has been hindered thus far by the lack of specific molecular markers. The Nd6 signal persists at the apex of the parasite for up to 5 min post-invasion, whereas the CRMPs signal at the apical tip of the parasite disappears [18]. No RDF3 signal was observed at the tip of the rhoptries even after 6 h post-invasion, indicating that replenishment at the tip occurs later, possibly concurrently with parasite multiplication. This dynamic localization can speak for a replenishment process as the one proposed for the MVs [16,17]. The dynamic of disappearance and replenishment have not been reported before for any proteins, and the precise mechanisms underlying the changes in localization of RDF3 and its temporal regulation during invasion warrant further investigation. U-ExM results showed that the staining of RDF3 was different from the luminal rhoptry bulb protein ROP5 and was visible as small punctate signal likely corresponding to vesicles decorating the rhoptry bulb. Collectively, the results suggest that RDF3 is not located inside the rhoptry bulb lumen but is likely present in vesicles that are delivered around the rhoptry bulb via vesicular trafficking. Subsequently, RDF3 may be replenished at the rhoptry tip during invasion. Colocalization between RDF3 and ICMAP2 firmly establishes that RDF3 localizes close to the AV. The apical localization of RDF3 is independent of the presence of the ICMTs, conoid, or rhoptries and remained invariably unaffected under perturbation of these structures. The bulbous localization of RDF3 is tightly associated with the organelle and always colocalizes with ROP5 under organelle positioning perturbations. Additionally, in SORTLR-depleted parasites, only the bulb localization disappeared confirming that this localization is dependent on the presence of the rhoptries. Intriguingly, RDF3 partially colocalizes with known rhoptry discharge factors CSCHAP and RASP2 without affecting their expression or trafficking. Similarly, RASP2 and CSCHAP do not influence RDF3 localization. Cryo-ET is a cutting-edge technique that has recently been used to dissect the structural arrangement of the RSA of Apicomplexa. Therefore, it was instrumental to use this technique to investigate if in the absence of RDF3, morphological changes had occurred in the apical complex structures. This analysis revealed that the core of the secretion machinery is not affected in RDF3-depleted parasites. However, given the presence of a smaller population of MVs and abundant extra vesicles in the conoid with the size and appearance of MVs, the absence of RDF3 likely perturbed the biogenesis and maintenance of the MVs. For example, the regulation of their trafficking may be disrupted, leading to the accumulation of numerous MV-like vesicles within the conoid. Moreover, it has been hypothesized that MVs serve as precursors to AVs, facilitating iterative rhoptry discharge [16,17]. More recently, a second AV and RSA have been observed in ICMAP2 knockdown parasites [17]. The mutant exhibits dispersed MVs that may be capable of becoming an AV if they prematurely reach the cell apex. In the RDF3 knockdown parasites, nearly 10% contained 2 AVs and 2 RSAs, indicating that the extra conoidal vesicles are indeed dysregulated MVs that prematurely became an AV with an RSA. Given the severity of the rhoptry discharge defect observed, which contrasts with the effect seen in ICMAP2-depleted parasites, RDF3 likely also plays a role in the function of the AV. To dissect the function of RDF3, we evaluated the localization and functional complementation of an RDF3 mutant with 3 strictly conserved residues among coccidian sequences downstream of the signal peptide. This RDF3-Mut1 affects RDF3 trafficking, with a punctate signal dispersed through the cytosol, yet the parasite was still able to form small plaques and invasion was restored to almost 30%. Additionally, in extracellular parasites RDF3-Mut1 accumulated at the apex. This accumulation can explain the slight rescue of the rhoptry discharge in this mutant. RDF3-Δ2 led to a severe decrease in RDF3 protein abundance indicating that the C-terminal region is important for the stability of the protein. Deletion of the hydrophobic region (RDF3-Δ1) was the most striking since it resulted in a severe defect in rhoptry discharge and invasion, while the protein was properly targeted. This indicates that the hydrophobic stretch is essential for RDF3 function but is not required for the protein’s localization or trafficking. Intriguingly, the apical localization of the nonfunctional RDF3-Δ1 persisted after invasion, whereas the functional WT RDF3 disappeared upon internalization of the parasite. In contrast, RDF3-Δ1 localization at the bulb disappeared just like the WT RDF3. In light of these results, it is tempting to speculate that the hydrophobic stretch plays a crucial role in RDF3 function at the tip. Functional RDF3 at the apex may be utilized during invasion, while RDF3 associated with the bulb, whether functional or not, may undergo degradation following invasion.

In summary, RDF3 emerges as a pivotal factor in rhoptry discharge, affecting MV biogenesis and likely influencing AV functionality. Its localization near the rhoptry bulb suggests a role in replenishing the new AV derived from MVs, potentially reloading the machinery for subsequent rhoptry discharge. While the hydrophobic region of RDF3 appears unrelated to protein trafficking and localization, it likely mediates interactions with crucial yet unidentified partners to facilitate AV function during invasion. This study unveils a dynamic process essential for effective rhoptry secretion, warranting further exploration.

## Materials and methods

### *Toxoplasma* and host cell culture

*T*. *gondii* tachyzoites RH strain, parental and modified strains, were grown on confluent monolayers of human foreskin fibroblasts (HFFs) in Dulbecco’s modified Eagle’s medium (DMEM, Gibco) with 5% fetal calf serum (FCS), 25 mg/ml gentamicin, and 2 mM glutamine. Parasites were grown 48 h in presence or in absence of either Rapamycin (50 nM), auxin (IAA) (500 μm), or ATc (1 μg/ml) prior analysis.

### Generation of parasite transgenic lines

For generation of all transgenic parasites, a specific guide RNA was designed to target the 3′ UTR of the gene of interest. Two universal primers containing the homology regions for the gene of interest were used to amplify by PCR the desired cassette. The *T*. *gondii* DiCre parental strain was used to generate RDF3-3Ty-U1 parasites. ΔKu80 RH strain was used to generate RDF3-2Ty strains. TiR1 strain was used to generate RDF3-mAID-3Ty parasites. To generate RDF3-3Ty-U1, we used the pG152-KI-3Ty-lox-SAG1-HXGPRT-U1 cassette. For the generation of RDF3-mAID-3Ty strain, we used the mAID-3Ty-HXGPRT plasmid. To generate RDF3-2Ty, we used pLinker-2xTy-HXGPRT plasmid. To tag RDF3 in Nd6-mAID-3HA, RASP2-mAID-3HA, CSCHAP-mAID-3HA, ICMAP2-mAID-3HA, and Myc-RNG2-mAID-3HA strains, we used the pLinker-2xTy-DHFR cassette. To tag RASP2 and CSCHAP in RDF3-3Ty-U1 parasites, we used the Myc3-LIC-DHFR plasmid.

To complement the RDF3-3Ty-U1 line with a wild-type copy of RDF3, a pRDF3-RDF3-4Myc sequence was cloned in the pUPRT plasmid. A total of 60 μg of the resulting vector pUPRT-pRDF3-RDF3-4Myc was digested and co-transfected using 15 μg of the pU6-Cas9-Universal-gRNAuprt vector [36]. Parasites that integrated pUPRT-pRDF3-RDF3-4Myc vector were selected using 5′-fluo-2′-deoxyuridine (FUDR) negative selection. To tag RDF3 in ARO-iKD-3Ty and SORTLR-mAID-3Ty strains, we transfected the RDF3 second copy (p-RDF3-RDF3-4Myc). Point mutagenesis introduced by Q5 site-directed mutagenesis (NEB) on pRDF3-RDF3-4myc were used to generate RDF3-Mut1 vector. RDF3-Δ1 was generated by deleting the 11 amino acids in the conserved hydrophobic region G113 ——A123 in the pUPRT-pRDF3-RDF3-4Myc vector. To generate RDF3-Δ2, we deleted the stretch of conserved charged residues (27 amino acids) in the C-terminal end D174 ——E200 in the pUPRT-pRDF3-RDF3-4Myc vector.

To transfect parasites with the corresponding plasmid, freshly egressed parasites were resuspended in Cytomix (10 mM KPO4, 120 mM KCl, 0.15 mM CaCl2, 5 mM MgCl2, 25 mM Hepes, 2 mM EDTA), supplemented with 3 mM ATP and 3 mM GSH and then mixed with the plasmid to be transfected and the corresponding gRNA. The DNA-parasite mixture is then subjected to an electric pulse (2,000 V, 50 ohms, and 25 μF) and transfected parasites were then incubated with a culture dish containing HFF in CO_2_ incubator at 37°C for maintenance and selection. Transgenic parasites were selected in the presence of mycophenolic acid (25 μg/ml) and xanthine (50 μg/ml) for the hypoxanthine-xanthine guanine-phosphoribosyl-transferase (HXGPRT) resistance cassette and in the presence of 1 μg/ml of pyrimethamine for the DHFR resistance. Stable strains were cloned by limiting dilution in 96-well plates and checked by PCR for genomic integration and analyzed by IFA and/or WB for protein expression.

### Western blot analysis

Freshly egressed parasites were pelleted by centrifugation at 1,100 g for 10 min. The pellet was washed in PBS and resuspended with SDS-PAGE (sodium dodecyl sulfate-polyacrylamide gel electrophoresis) loading buffer (+/− 10 mM DTT) and heated at 95°C for 10 min (for transmembrane proteins, the heating step was skipped). Proteins separated by SDS-PAGE were transferred to nitrocellulose membrane and immunoblot analysis was performed. Primary and secondary antibodies are diluted in 5% milk/0.05% Tween/PBS and washes are performed in 0.05%Tween/PBS. Proteins were visualized with Pierce ECL western blotting substrate according to the manufacturer’s protocol.

### Immunofluorescence analysis

Freshly egressed *T*. *gondii* tachyzoites were used to infect HFF monolayers on coverslips and incubated at 37°C for 24 h prior fixation, and 4% paraformaldehyde (PFA)/0.05% glutaraldehyde (GA) was used to fix the infected cells prior quenching with 0.1 M glycine/PBS. First, cells were permeabilized and blocked with 2% BSA/0.2% Triton/PBS for 1 h (in the case of methanol fixation, only blocking with 2% BSA/PBS for 1 h was performed). After blocking, cells were probed with primary antibodies diluted in 2% BSA/0.2% Triton/PBS for 1 h followed by 3 washes with PBS. Then, cells were incubated with secondary antibodies diluted in 2% BSA/0.2% Triton/PBS for 1 h followed by 3 washes with PBS. DAPI (4′,6-diamidino-2-phenylindole; 50 mg/ml in PBS) was used to stain the parasites and HFF nuclei and coverslips were mounted on Fluoromount G (Southern Biotech) on glass slides. Images were taken by LSM700 confocal microscope (Zeiss) at the bioimaging core facility of the Faculty of Medicine at the University of Geneva. Images were processed and analyzed using ImageJ software.

### Plaque assay

Confluent monolayer of HFF were infected with freshly egressed parasites and incubated for 7 days in presence or absence of drug, and 4% PFA/0.05% glutaraldehyde was used to fix the parasites for 10 to 15 min. After neutralization with 0.1 M glycine/PBS, cells were stained using crystal violet.

### Invasion assay

Freshly egressed parasites, pretreated for 48 h with the corresponding drug, were diluted 1:10, inoculated on HFF coverslips, and centrifuged for 30 s at 1,200 g to let them settle down on the host cell layer. Parasites were incubated for 30 min at 37°C to allow them to invade prior fixation using PFA/Glu for 7 min. Parasites were first incubated with anti-SAG1 antibody in nonpermeabilized conditions (2% PBS-BSA) to stain extracellular parasites only. After several washes with PBS (3 or more), cells were fixed using 1% formaldehyde/PBS for 7 min under fume hood followed by a wash with PBS. Next, cells were permeabilized with 0.2% Triton/PBS and stained with anti-GAP45 antibody (staining both intracellular and extracellular parasites). Secondary antibodies were used as previously described. A total of 100 parasites were counted for each experiment, and % of intracellular parasite (GAP45 positive only) relative to the total number of parasites (GAP45+ + GAP45+/SAG1+ parasites) is presented. Results are mean ± standard deviation of 3 independent biological replicate experiments.

### Induced egress assay

Freshly egressed parasites pretreated 24 h +/− rapamycin were used to infect HFF monolayers. The coverslips were washed 2 h postinfection to remove non-invading extracellular parasites and then left to grow for 36 h at 37°C. Prior to inducing egress, the cells were washed 3 times with egress buffer (serum-free DMEM) to remove the serum. The infected HFF cells were then incubated for 7 min at 37°C with serum-free DMEM containing either BIPPO [37] or DMSO as a control. Prior to fixing with PFA/GLU, the monolayers of the WT positive control were observed under the microscope to check if the parasites had egressed. IFA was performed as previously described using anti-GAP45 antibody to stain the parasites and anti-GRA3 antibody to stain the PV. A total of 100 vacuoles were counted per condition and scored as egressed or non-egressed for quantification of the percentage of egress. Results are mean ± standard deviation of 3 independent biological replicates. The control experiment with DMSO showed no egress.

### Intracellular growth

Freshly egressed parasites pretreated 48 h +/− rapamycin were used to infect HFF monolayers cells and the coverslips were washed 2 h postinfection. Infected cells were fixed 24 h postinfection with PFA/glutaraldehyde. IFAs were performed using anti-GAP45 antibodies to detect parasites. The number of parasites per vacuole was scored, counting 100 vacuoles for each condition. Results are mean ± standard deviation of 3 independent biological replicates.

### E-vacuole secretion assay

Rhoptry secretion was assessed using e-vacuole detection assay [28]. Freshly egressed parasites pretreated with the corresponding drug, were first counted (number of parasites/ml), washed in PBS, and resuspended in prechilled DMEM medium containing ±1 μm Cytochalasin D (use to inhibit parasite entry without impacting on rhoptry secretion) followed by incubation on ice for 10 min. The parasites were then added to prechilled HFF-coated coverslips and incubated for 20 min on ice. The coverslips are then washed with cold PBS before adding complete DMEM medium containing ±1 μm Cytochalasin D or DMSO, for controls, and further incubated for 20 min at 37°C in the water bath. Approximately 4% paraformaldehyde was used to fix the parasites for 15 min. IFA was performed as described before. For this assay, ROP1 was used as a marker for rhoptry secretion (visualization of the e-vacuoles), while GAP45 was used to stain the parasites’ pellicles, and 100 parasites were analyzed per experiment for rhoptries secretion and quantified for the presence of the apical tip staining. The results are shown as mean ± standard deviation of 3 independent biological replicate experiments.

### STAT6-P as marker for rhoptry secretion

Freshly egressed parasites were incubated with HFFs for 15 min at 37°C in Endo Buffer (106 mM sucrose, 10 mM Mg_2_SO_4_, 44.7 mM K_2_SO_4_, 20 mM Tris (pH 8.2), 5 mM glucose, 3.5 mg/ml BSA) known to inhibit invasion. Next, parasites were incubated in pre-warmed serum-free DMEM for 5 min at 37°C to initiate parasites invasion prior to fixation with 100% ice-cold methanol for 10 min. DiCre and RDF3 parasites were pretreated 48 h +/− rapamycin for this experiment. Parasites from the tet-inducible knockdown strain of ASP3 (ASP3-iKD), pretreated 48 h with ATc, were used as negative control as they are defective in rhoptry secretion following ATc treatment [30]. IFA was done using anti-STAT6-P antibody and the number of STAT6-P positive host cell nuclei were counted compared to the total number of cells for all conditions. A total of 100 nuclei were counted for each condition and the experiment was done in 2 independent biological replicates.

### Secreted RON4

To distinctly assess the discharge of RON4, the same procedure as the e-vacuole secretion assay was used and then the cells were permeabilized with 0.1% saponin. IFAs were performed using α-RON4 (dilution 1:10) and α-GAP45 antibodies (dilution 1:10,000) in 2% BSA/PBS, and 100 parasites per experiment were counted and RON4 secretion quantified based on the presence of staining at the apical tip of the parasite. Results are a mean ± standard deviation of 3 independent biological replicates.

### Cytochalasin D treatment on extracellular parasites

Freshly egressed parasites pretreated 48 h +/− rapamycin or IAA or ATc were first washed in PBS and resuspended in warm PBS containing ±1 μm Cytochalasin D followed by incubation on ice for 10 min. The parasites were incubated for 30 min at 37°C in the water bath. Approximately 4% paraformaldehyde was used to fix the parasites for 7 min. IFA was performed as described before.

### Proteinase K protection assay

Freshly egressed *T*. *gondii* tachyzoites were resuspended in 3 ml cold SoTE (0.6 M sorbitol, 20 mM Tris–HCl (pH 7.5), and 2 mM EDTA) and split into 5 tubes (0.5 ml each). Cold SoTE was added to tube 1 as a control. Tubes 2 to 5 were permeabilized with 0.5 ml cold SoTE/digitonin (Sigma) with different concentrations of digitonin (0.01% (tube2), 0.01% (tube3), 0.005% (tube4), and 0.0025% (tube5)). Samples were carefully mixed by inversion and incubated on ice/10 min prior to centrifugation (2,000 3 g/4C/10 min). Supernatant was discarded, and 0.5 ml cold SoTE was added to tube 1, while 0.5 ml of cold Proteinase K (Sigma, 10 mg/ml)/SoTE was added to tubes 3 to 5. All tubes were gently inverted and incubated on ice/30 min. Proteinase K was inactivated by addition of cold trichloroacetic acid to a final concentration of 10% on ice/30 min. Samples were centrifuged (14,000 rpm/20 min), washed with acetone, air dried, and resuspended in TE prior to SDS-PAGE.

### Solubility assay

Freshly egressed parasites, pretreated for 48 h +/− rapamycin, were pelleted and resuspended in PBS, PBS containing NaCl, PBS triton (1% Tx), or PBS containing Na_2_CO_3_. These samples are then freeze and thawed using liquid nitrogen and 37°C water bath. The pellet and the soluble fraction are separated by centrifugation for 30 min at 4°C max speed. Samples were finally resuspended with SDS-PAGE loading buffer (+/− 10 mM DTT) and heated at 95°C for 10 min.

### Serial section transmission electron microscopy (ssTEM)

HFF cells were infected with *T*. *gondii* parasites and let to grow for 24 h before fixing with 2% PFA and 2.5% GA in 0.1 M sodium cacodylate buffer (pH 7.4) for 1 h at room temperature. The infected cells were then washed with 0.1 M sodium cacodylate buffer (pH 7.4) and fixed with 1.5% potassium ferrocyanide and 1% osmium tetroxide in 0.1 M sodium cacodylate buffer (pH 7.4) for 1 h followed by 1% osmium tetroxide in 0.1 M sodium cacodylate buffer (pH 7.4) for another hour. Cells are then washed in double-distilled water twice and stained with aqueous 1% uranyl acetate for 1 h. Cells are washed with double-distilled water twice, and then dehydrated in graded ethanol series (2 × 50%, 70%, 90%, 95%, and 2 × absolute ethanol) for 10 min each. Cells are infiltrated with a graded series of Durcupan resin diluted with ethanol at 1:2, 1:1, and 2:1 for 30 min each and then twice with pure Durcupan for 30 min each. Cells were infiltrated with fresh Durcupan resin for an additional 2 h. A coverslip with cells was placed on a 1-mm high silicone ring filled with fresh resin and placed on a glass slide coated with mold-separating agent. It was then polymerized for 24 h at 65°C. Afterwards, the coverslip was removed from the resin disk by putting it in hot water (60°C) and then liquid nitrogen. A laser microdissection microscope was used to outline the position of the PV on the exposed surface of the resin. The area is then cut out from the disk and glued to a blank resin block. Using a Leica Ultracut UCT microtome (Leica Microsystems) and a glass knife the cutting face was trimmed. Then, a 70-nm ultrathin serial sections were cut with a diamond knife (DiATOME) and collected onto 2-mm single-slot copper grids (Electron Microscopy Sciences) coated with Formvar plastic support film. Sections were examined using a Tecnai 20 TEM operating at an acceleration voltage of 80 kV and equipped with a side-mounted MegaView III CCD camera (Olympus Soft-Imaging Systems) controlled by iTEM acquisition software (Olympus Soft-Imaging Systems).

### Conoid extrusion assay by IFA for *Toxoplasma*

Freshly egressed tachyzoites were incubated with 2 μm CytD or DMSO for 30 min. Parasites were then seeded on gelatin-coated coverslips, and warm media containing BIPPO was added on top. Coverslips were centrifuged at 1,000 r.p.m. for 1 min and incubated for 10 min at 37°C. Parasites were then fixed and stained, with anti-Ty and anti-methylated lysine antibodies (3Me-Pan).

### Cryo-expansion microscopy

Cryo-expansion microscopy preparation was performed as previously described with few timesaving modifications [38,39]. Briefly, extracellular parasites (in PBS supplemented with BIPPO to induce conoid extrusion) seeded on Poly-D-Lysin-coated coverslips were fixed using cryofixation using conventional rapid plunging in liquid ethane-propane then incubated in acetone precooled with liquid nitrogen and placed on dry ice overnight. This allows the dry ice evaporation and a gradual increase in the sample temperature from −180°C to 0°C. The water of the sample is replaced by acetone that preserves the sample architecture. We then rehydrate the sample using ethanol mixed with water and proceed to the U-ExM protocol. Cells were embedded in an Temed/APS/Monomer solution (19% Sodium Acrylate, 0,1% BIS-AA, and 10% AA in PBS 10×) gel on ice, prior incubation at 37°C during 30 min. Gels were then denatured at 95°C during 1 h 30 with denaturation buffer (200 mM SDS; 200 mM NaCl; 50 mM Tris-Base, pH 9). The gels were then expanded 2 times 30 min minimum in water. Gels were then measured before shrinking (2 washes of 15 min in PBS) and cutting in small piece of gel (roughly 1 cm × 1 cm). The piece of gel was then incubated with primary antibodies in 2% BSA/PBS for 3 h. The gels were then washed 3 times in PBS during 10 min. Then, followed the incubation with secondary antibodies in 2% BSA/PBS for 3 h. Gels were then washed 3 times in PBS for 10 min before final expansion (2 times 30 min in water). Imaging was performed using a Leica TCS SP8 inverted microscope, equipped with an HC PL Apo 100×/1.40 Oil CS2 objective and with HyD detectors. Pieces of gels were cut and placed on a 24 mm poly-lysine–coated coverslip (to avoid gel drifting during acquisition) fitted in a metallic O-ring 35 mm imaging chamber (Okolab). Z-stack was acquired with the Leica LAS X software and deconvolved with the built-in “Lightning” mode. Images were then processed with the ImageJ software.

### Immuno-electron microscopy (immuno-EM)

Cultured HFF cells infected with *T*. *gondii* RDF3-2Ty strain were scrapped and pelleted. Cell pellet inside 300 μl carriers coated with soy lecitine in chloroform was high-pressure frozen using Leica EM ICE High pressure freezing machine. Carrier halfs containing frozen sample were transferred into Leica EM AFS automated freeze-substitution device. High-pressure freezing and freeze-substitution was performed at DCI Geneva Cryogenic Facility, Science II, University of Geneva. From polymerized block, the metal carrier was removed by warming to 40°C and cooling in liquid nitrogen. Exposed cell pellet was cut by ultramicrotome (Leica Ultracut UCT) and diamond knife (DiTOME) and 60 nm ultrathin sections were collected on the parlodion-coated 2 mm slot grids (SynapTek DOT, Ted Pella). Post-embedding immunolabeling was performed by incubation on drops of on parafilm in wet chamber as following: incubation with blocking buffer (2% BSA with 0.02% Tween20 in 0.01 M PBS) 3 times for 10 min each, incubation with primary antibody (anti-Ty) diluted in blocking buffer for 1 h, following 3 washes with blocking buffer for 10 min each, and finally incubated with secondary antibody Goat anti-mouse coupled with 10 nm colloidal gold (GAM-10nm) at dilution 1:20 for 1 h. After immunolabeling, grids with sections were washed twice for 10 min each with blocking buffer, following by PBS for 3 times for 5 min, fixed bound Abs with 1% glutaraldehyde in water for 3 min, washed with water 3 times for 2 min each and finally post-stained with 1% aqueous uranyl acetate for 3 min. Samples were analyzed and imaged by Tecnai 12 G2 transmission electron microscope operating at 80 kV acceleration voltage, equipped with MegaView III side-mounted CCD camera controlled with iTEM image acquisition software (Olympus-Soft Imaging Solution). Ultramicrotomy and TEM imaging were performed at Electron Microscopy Facility (PFMU) at the University Medical Centre (CMU), Medical Faculty, University of Geneva.

### Cryo-electron tomography (cryo-ET)

Cryo-ET was performed on a Thermo Fisher Krios G3i 300 keV field emission cryo-transmission electron microscope. Dose-fractionated imaging was performed using the SerialEM software [40] on a K3 direct electron detector [41] (Gatan) operated in electron-counted mode. Motion correction of images was done using the Alignframe function in IMOD [42]. Imaging was done using a Volta phase plate [43] to increase contrast without high defocus, and the Gatan Imaging Filter (Gatan) with a slit width of 20 eV to increase contrast by removing inelastically scattered electrons [44]. After initially assessing cells at lower magnifications for suitability of ice thickness and plasma membrane integrity, tilt series were collected with a span of 120° (−60° to +60°; bidirectional scheme) with 2° increments at a magnification of 33,000× (with a corresponding pixel size of 2.65 Å) and a defocus range of −1 to −4 μm. Each tilt series was collected with a cumulative dose of around 140 e^–^ Å^−2^. Once acquired, tilt series were aligned using the 10 nm colloidal gold as fiducials and reconstructed into tomograms by our in-house automated computation pipeline utilizing the IMOD software package [42].

### Quantification and analysis of cryo-ET data

We obtained a total of 46 RDF3 knockdown and 37 RDF3 control tomograms. For analysis, after filtering for intact parasites and adequate contrast, tomograms were selected randomly. For wild-type *T*. *gondii*, tomograms were randomly selected from a previously collected data set [10]. For quantification of cellular features, IMOD models were generated on the following features within 20 RDF3 knockdown, 4 RDF3 control, and 6 wild-type *T*. *gondii* tomograms: (1) the AV; (2) MVs; (3) ICMTs; and (4) extra vesicles width within the conoid. Values for vesicle numbers, dimensions, and distances were extracted using a custom Python script. To determine statistical significance, the two-sided *T* test was performed between 2 populations with normal distributions, and the two-way Mann–Whitney U test was used for populations with non-normal distributions.

### Segmentation of Cryo-ET data

RDF3-knockdown parasites were manually segmented in IMOD and subsequently imported into UCSF ChimeraX for generation of movies [45].

## Supporting information

S1 Fig Strategy for gene tagging and down-regulation. (A) Genetic strategy used to generate inducible-knockdown strains and integration PCR on clonal lines and DiCre parental line to assess the excision of the cassette in presence of rapamycin (−Rapa = 3,125 bp; +Rapa = 900 bp). (B) Western blots using anti-Ty antibodies showing the down-regulation of the tagged proteins compared to the parental line DiCre. Catalase (anti-CAT) is used as a loading control. Source data are provided as S1 Data.
(TIF)

S2 Fig Generating RDF3-2Ty strain. (A) Integration PCR on the RDF3-2Ty and RH parental strain with primers P1/P6 (800 bp). (B) Quantification of the western blots assessing the expression level of RDF3. A parametric paired *t* test was used to assess significance; the two-tailed *p*-values are written on the graphs. (C) Retraction/extrusion assay by regular IFA. White arrowhead, extruded conoid; black arrowhead, retracted conoid. Anti-Ty antibody (green) is used to stain RDF3. Anti-methylated lysine antibody (3Me-Pan) is used to stain the apical cap and the conoid. Scale bar = 2 μm. (D) Immunofluorescence of RDF3-2Ty intracellular parasites using anti-Ty antibody (green), anti-RON9 and anti-IMC1 antibody (magenta). Scale bar = 2 μm. Source data are provided as S1 Data.
(TIF)

S3 Fig Expression and localization of the complemented strain. (A) Integration PCR for RDF3-4Myc strain performed with primers P5/P6 (5′ integration = 3,300 bp), and P7/P8 (3′ integration = 2,600 bp). (B) Western blot using anti-Myc antibodies showing the regulation of the complemented strain compared to the parental line DiCre, RDF3-3Ty-U1, and the parental strain complemented with a second copy of RDF3 as a control. Catalase (anti-CAT) is used as a loading control. (C) Immunofluorescence of RDF3-4Myc parasites using anti-Myc antibody (green) and anti-ARO antibody (magenta). DAPI (blue). Scale bar = 2 μm. Source data are provided as S1 Data.
(TIF)

S4 Fig Rhoptry proteins expression and localization in absence of RDF3. (A) Immunofluorescence using anti-ARO (magenta), anti-ROP5, and anti-RON9 antibodies (green) that shown a normal localization of rhoptry proteins in absence of RDF3. Counter-staining of DNA with DAPI (blue). Scale bar = 2 μm. (B) Western blots showing a normal expression of ROP5 and RON9 in absence of RDF3. Catalase (anti-CAT) is used as a loading control. Source data are provided as S1 Data.
(TIF)

S5 Fig Tagging of RDF3 with the mini-AID cassette. (A) Quantification of invasion of parasites at different time points post-invasion (*n* = 3 biologically independent experiments). (B) Western blot showing the expression of RDF3-2Ty at different time points. (C) Cell cycle transcriptomic profile of RDF3. Cell cycle was compared to RON4 and ROP7 for control. (D) Immunogold labeling of RDF3-2Ty. Left panel shows longitudinal section through apical part of the parasites, right panel shows transversal section through the bundle of rhoptries. Gold particles location is highlighted with red arrowheads. rho: rhoptries. Scale bar = 500 nm. (E) Western blot showing the absence of down-regulation of RDF3 in presence of IAA. Catalase (CAT) is used as a loading control. (F) Immunofluorescence of intracellular RDF3-mAID-3Ty parasites (±IAA). DAPI (blue). Scale bar = 2 μm. (G) Plaque assay showing that depletion of RDF3 using the mAID system does not affect parasite survival. Parental strain TiR1 was used as a control. Image representative of 3 biologically independent experiments. (H) Immunofluorescence of extracellular RDF3-mAID-3Ty parasites (±IAA) treated with cytochalasin D (+CytD). DAPI (blue). Scale bar = 2 μm. (I) Solubility assay of RDF3-3Ty-U1. P = pellet. S = supernatant. Catalase (cytoplasmic protein) is used as a control soluble in all conditions. GAP45 (anchored to IMC and PM) is used as a control only soluble by Tx-100. Source data are provided as S1 Data.
(TIF)

S6 Fig Immunofluorescences showing that RDF3 apical localization is independent from rhoptries. (A) Immunofluorescence showing the localization of RDF3 and ICMAP2 in intracellular parasites (±IAA). DAPI (blue). Scale bar = 2 μm. (B) Immunofluorescence on extracellular parasites (+CytD) using anti-ROP5 and anti-GAP45 antibodies to localize RDF3 in presence or absence of ICMAP2. DAPI (blue). Scale bar = 2 μm. (C) Immunofluorescence showing the localization of RDF3 and RNG2 in intracellular parasites (±IAA). DAPI (blue). Scale bar = 2 μm. (D) Immunofluorescence on extracellular parasites (+CytD) using anti-ROP5 and anti-GAP45 antibodies to localize RDF3 in presence or absence of RNG2. DAPI (blue). Scale bar = 2 μm. (E) Immunofluorescence showing the localization of RDF3 and ARO in intracellular parasites (±ATc). DAPI (blue). Scale bar = 2 μm. (F) Immunofluorescence on extracellular parasites (+CytD) using anti-ROP5 and anti-GAP45 antibodies to localize RDF3 in presence or absence of ARO. DAPI (blue). Scale bar = 2 μm. (G) Immunofluorescence showing the localization of RDF3 and SORTLR in intracellular parasites (±IAA). DAPI (blue). Scale bar = 2 μm. (H) Immunofluorescence on extracellular parasites (+CytD) using anti-ROP5 and anti-GAP45 antibodies to localize RDF3 in presence or absence of SORTLR. DAPI (blue). Scale bar = 2 μm. Source data are provided as S1 Data.
(TIF)

S7 Fig Localization of RDF3, RASP2, and ICMAP2 are independent. (A) Immunofluorescence showing the mislocalization of RDF3 second copy when tagged with the MiniTurbo cassette. (B) Immunofluorescence to assess RDF3 localization with Nd6, RASP2, and CSCHAP in intracellular parasites. Counter-staining of DNA with DAPI (blue). Scale bar = 2 μm. (C) Immunofluorescence to assess RASP2 and CSCHAP localization in absence of RDF3 in extracellular parasites (+CytD). Counter-staining of DNA with DAPI (blue). Scale bar = 2 μm. Staining observed at the basal pole of the parasite is due to a nonspecific labeling of the anti-Myc rabbit antibody. (D) Immunofluorescence showing the localization of RASP2 and CSCHAP 15 min post-invasion. Counter-staining of DNA with DAPI (blue). Scale bar = 2 μm. Source data are provided as S1 Data.
(TIFF)

S8 Fig Comparison of apical organelles in wild-type vs. RDF3 knockdown parasites. 2D slice of a tomogram through a wild-type (left) and RDF3 knockdown cell (right), with and without color overlay, displaying (A) 2 rhoptries docked at the AV, and (B) the MVs along the ICMT as well as any extra vesicles in the conoid.
(TIF)

S9 Fig Functional dissection of RDF3. (A) Integration PCR on RDF3 mutants, RDF3-depleted parasites and DiCre parental line performed with primers P7/P8 (5′ integration = 3,300 bp) and P9/P10 (3′ integration = 2,600 bp). (B). Western blot using anti-Ty antibodies showing the regulation of RDF3-3ty-U1 in the complemented mutated strains. Catalase (anti-CAT) is used as a loading control. (C) Solubility assay of RDF3 mutants. P = pellet. S = supernatant. Catalase (cytoplasmic protein) is used as a control soluble in all conditions. (D) Quantification of plaque assays for DiCre, RDF3-3Ty-U1, and the 3 RDF3 mutants’ strain (±Rapa). (Mean ± SD; *n* = 3 biologically independent experiments.) Statistical significance was assessed by a two-way ANOVA significance with Tukey’s multiple comparison. (E) Intracellular replication assay. Graph representing the number of parasites per vacuole observed at 36 h post-invasion. (Mean ± SD; *n* = 3 biologically independent experiments.) Source data are provided as S1 Data.
(TIF)

S1 Movie Tomogram of an RDF3 knockdown cell (+Rapa), slicing through the apical end to reveal the 3D segmentation of the PPM (blue-gray), ICMTs (yellow), AV (pink), rhoptries (orange), MVs (cyan), and extra vesicles (red). (MOV)

S2 Movie Tomogram of an RDF3 knockdown cell (+Rapa), slicing through the apical end to reveal the 3-D segmentation of the PPM (blue-gray), ICMTs (yellow), AV (pink), rhoptries (orange), MVs (cyan), and extra vesicles (red). (MOV)

S3 Movie Tomogram of an RDF3 non knockdown cell (−Rapa), slicing through the apical end to reveal the 3D segmentation of the PPM (blue-gray), ICMTs (yellow), AV (pink), rhoptries (orange), MVs (cyan), and extra vesicles (red). (MOV)

S4 Movie Tomogram of a WT cell (parental strain), slicing through the apical end to reveal the 3D segmentation of the PPM (blue-gray), ICMTs (yellow), AV (pink), rhoptries (orange), MVs (cyan), and extra vesicles (red). (MOV)

S1 Data Source data file with all numerical input data for graphs shown in figures. (XLSX)

S2 Data Primers and antibodies used in this study. (XLSX)

S1 Raw Images Raw images of uncropped DNA agarose gels and western blots. (PDF)

## Abbreviations

AV: apical vesicle
CRMP: cysteine repeats modular protein
cryo-ET: cryo-electron tomography
DMEM: Dulbecco’s modified Eagle’s medium
EV: extra vesicle; FCS, fetal calf serum
GA: glutaraldehyde
HFF: human foreskin fibroblast
IAA: indole-3-acetic acid
ICMAP2: intraconoidal microtubules associated protein 2
ICMT: intraconoidal microtubule
IFA: immunofluorescence assay
IMC: inner membrane complex
LOPIT: Localization of Organelle Proteins by Isotope Tagging
mAID: mini auxin-induced degron
MV: microtubule-associated vesicle
PFA: paraformaldehyde
RASP2: rhoptry apical surface protein 2
RDF3: rhoptry discharge factor 3
RSA: rhoptry secretion apparatus
SDS-PAGE: sodium dodecyl sulfate-polyacrylamide gel electrophoresis
ssTEM: serial section transmission electron microscopy
UTR: untranslated region
