# Supplementary figures and images for: Toxoplasma gondii rhoptry discharge factor 3 is essential for invasion and microtubule-associated vesicle biogenesis
Source: PLoS Biol. 2024 Aug 13;22(8):e3002745. doi: 10.1371/journal.pbio.3002745 (PMC11343613; doi:10.1371/journal.pbio.3002745)

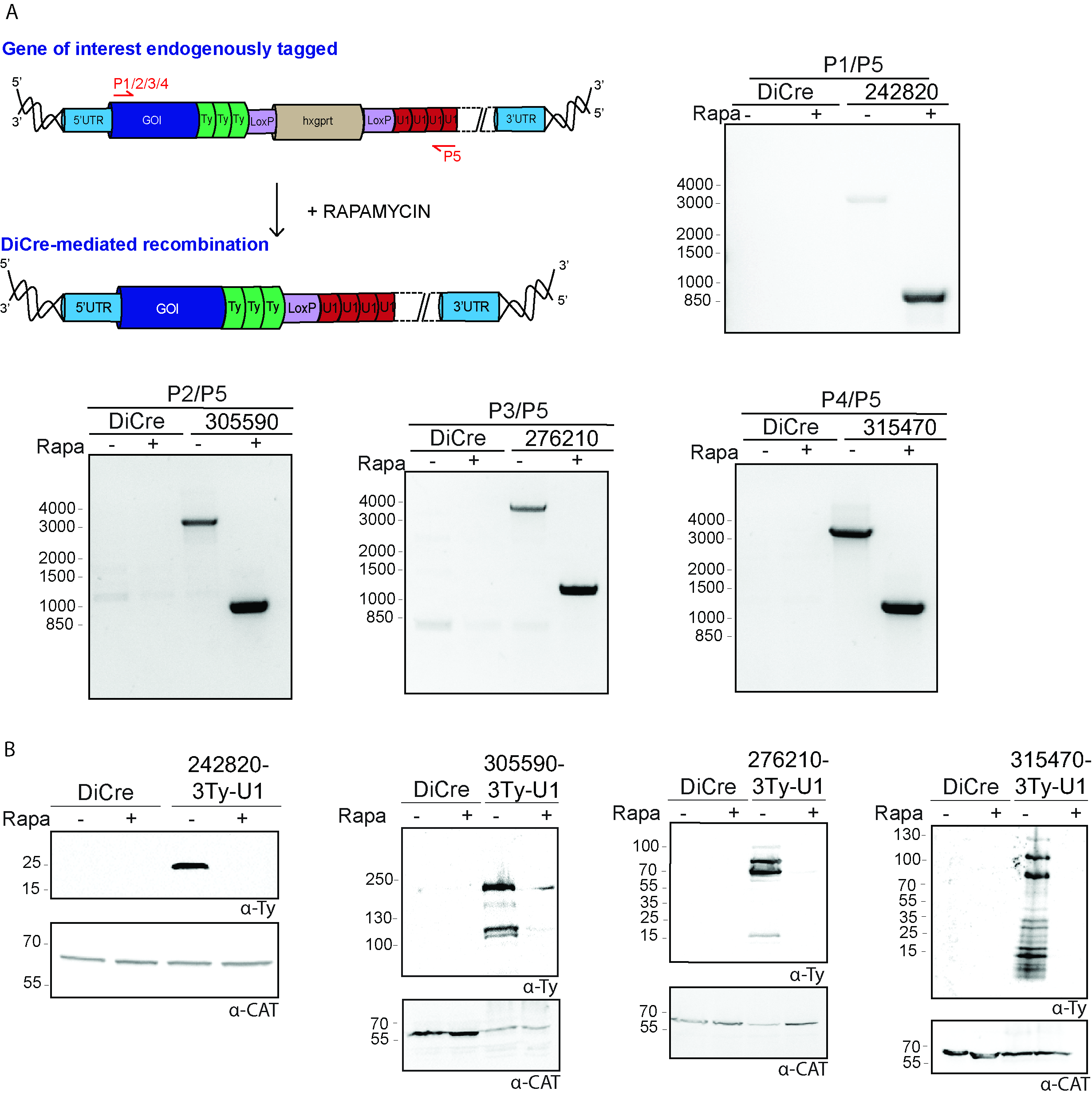

Supplement: S1 Fig — (A) Genetic strategy used to generate inducible-knockdown strains and integration PCR on clonal lines and DiCre parental line to assess the excision of the cassette in presence of rapamycin (−Rapa = 3,125 bp; +Rapa = 900 bp). (B) Western blots using anti-Ty antibodies showing the down-regulation of the tagged proteins compared to the parental line DiCre. Catalase (anti-CAT) is used as a loading control. Source data are provided as S1 Data. (TIF) [file pbio.3002745.s001.tif]

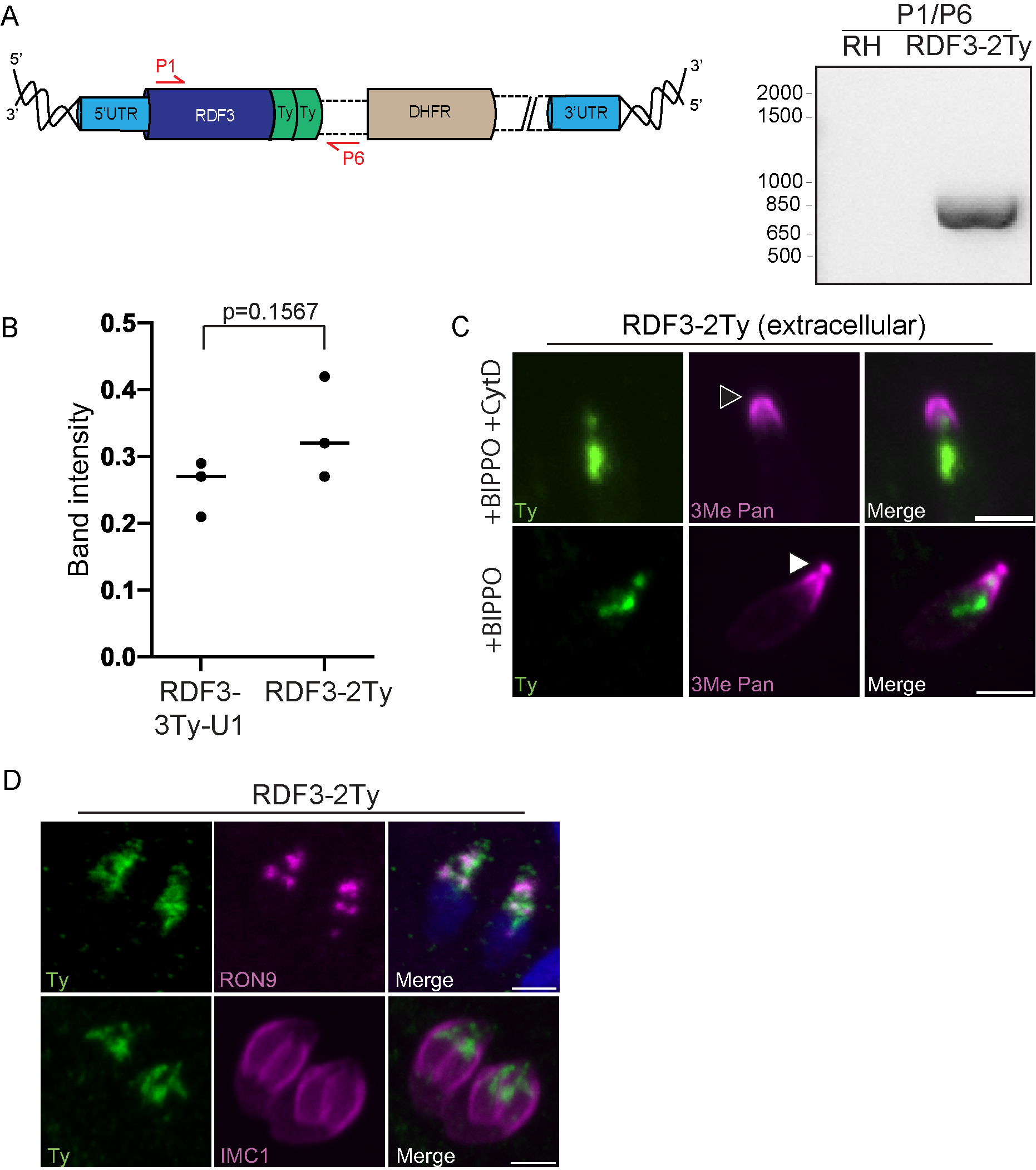

Supplement: S2 Fig — (A) Integration PCR on the RDF3-2Ty and RH parental strain with primers P1/P6 (800 bp). (B) Quantification of the western blots assessing the expression level of RDF3. A parametric paired t test was used to assess significance; the two-tailed p-values are written on the graphs. (C) Retraction/extrusion assay by regular IFA. White arrowhead, extruded conoid; black arrowhead, retracted conoid. Anti-Ty antibody (green) is used to stain RDF3. Anti-methylated lysine antibody (3Me-Pan) is used to stain the apical cap and the conoid. Scale bar = 2 μm. (D) Immunofluorescence of RDF3-2Ty intracellular parasites using anti-Ty antibody (green), anti-RON9 and anti-IMC1 antibody (magenta). Scale bar = 2 μm. Source data are provided as S1 Data. (TIF) [file pbio.3002745.s002.tif]

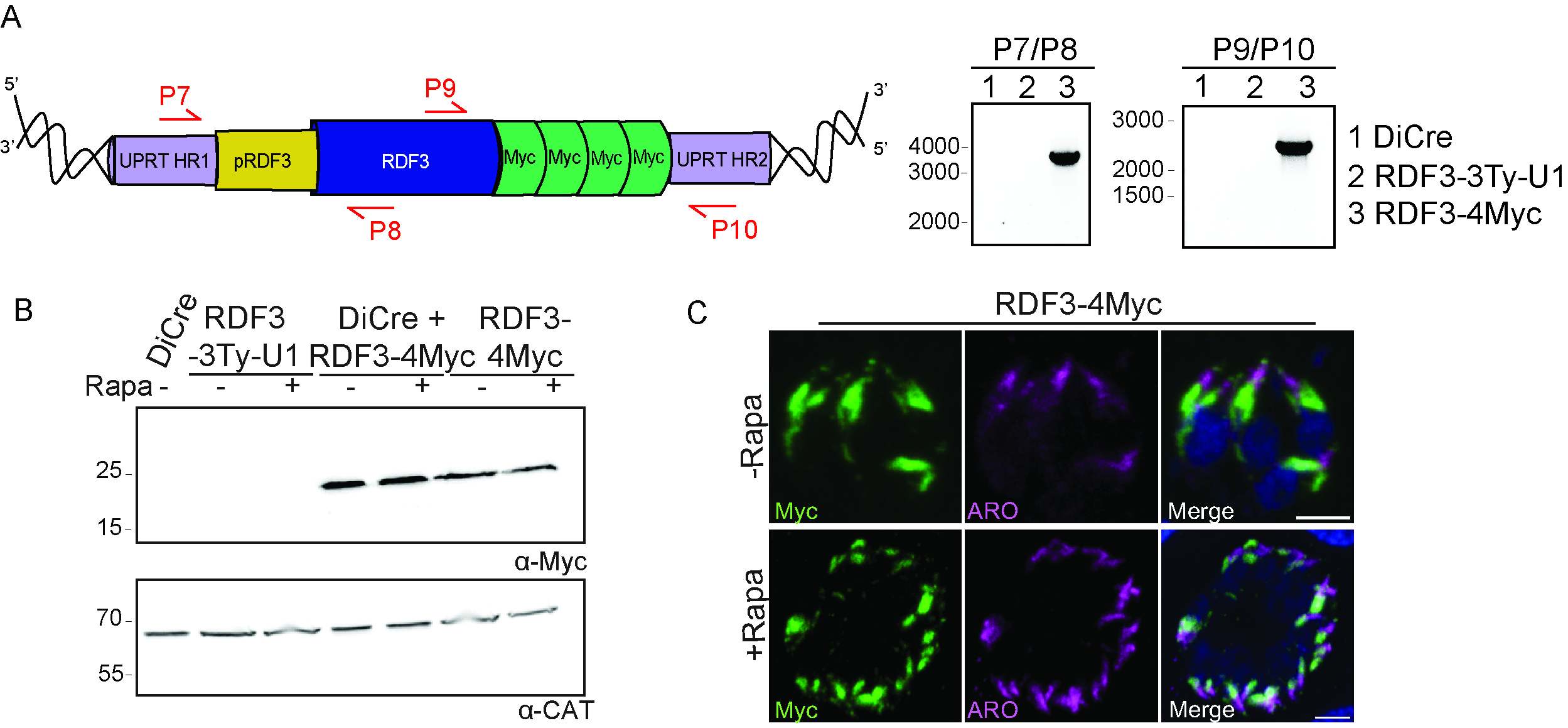

Supplement: S3 Fig — (A) Integration PCR for RDF3-4Myc strain performed with primers P5/P6 (5′ integration = 3,300 bp), and P7/P8 (3′ integration = 2,600 bp). (B) Western blot using anti-Myc antibodies showing the regulation of the complemented strain compared to the parental line DiCre, RDF3-3Ty-U1, and the parental strain complemented with a second copy of RDF3 as a control. Catalase (anti-CAT) is used as a loading control. (C) Immunofluorescence of RDF3-4Myc parasites using anti-Myc antibody (green) and anti-ARO antibody (magenta). DAPI (blue). Scale bar = 2 μm. Source data are provided as S1 Data. (TIF) [file pbio.3002745.s003.tif]

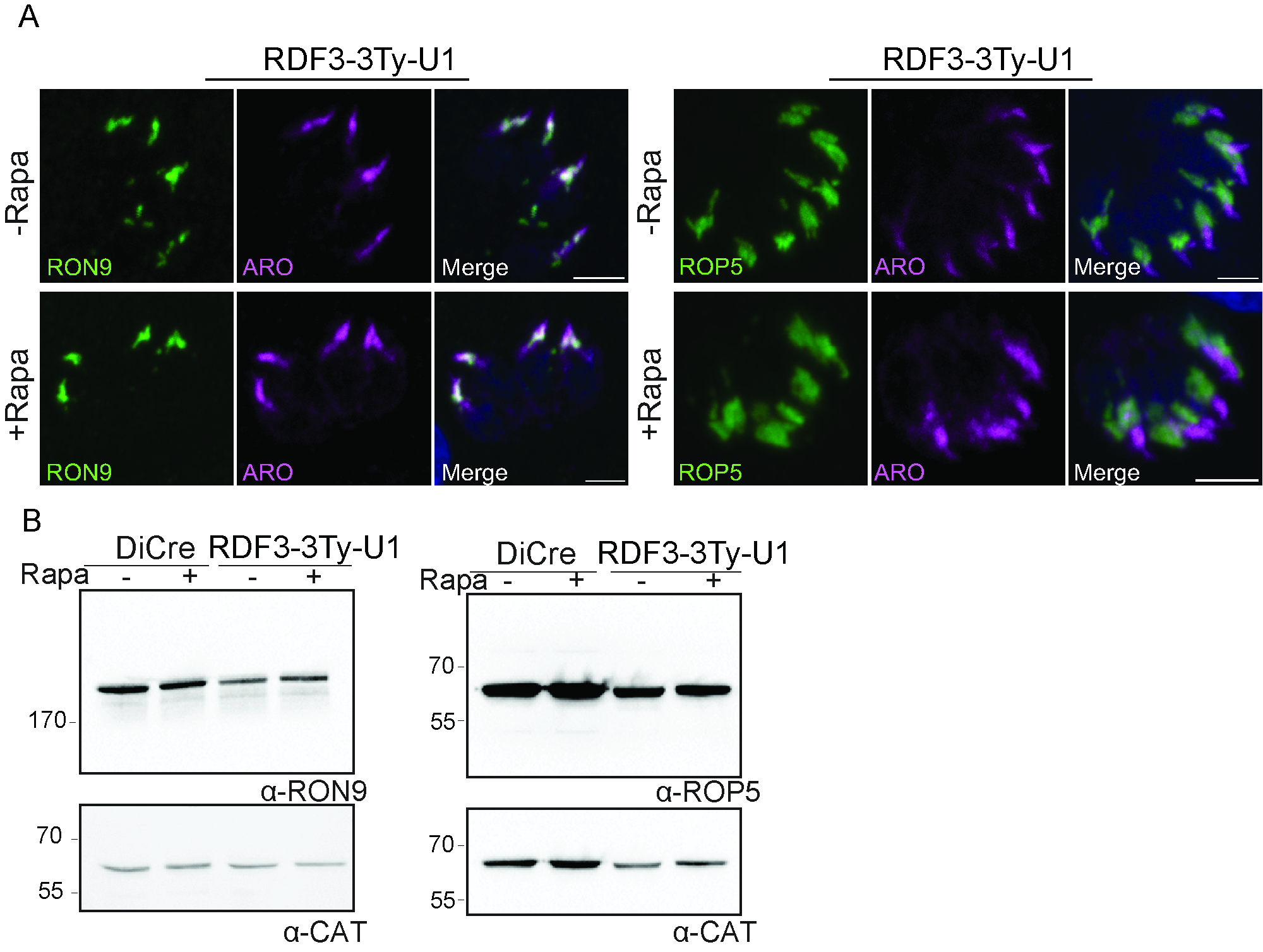

Supplement: S4 Fig — (A) Immunofluorescence using anti-ARO (magenta), anti-ROP5, and anti-RON9 antibodies (green) that shown a normal localization of rhoptry proteins in absence of RDF3. Counter-staining of DNA with DAPI (blue). Scale bar = 2 μm. (B) Western blots showing a normal expression of ROP5 and RON9 in absence of RDF3. Catalase (anti-CAT) is used as a loading control. Source data are provided as S1 Data. (TIF) [file pbio.3002745.s004.tif]

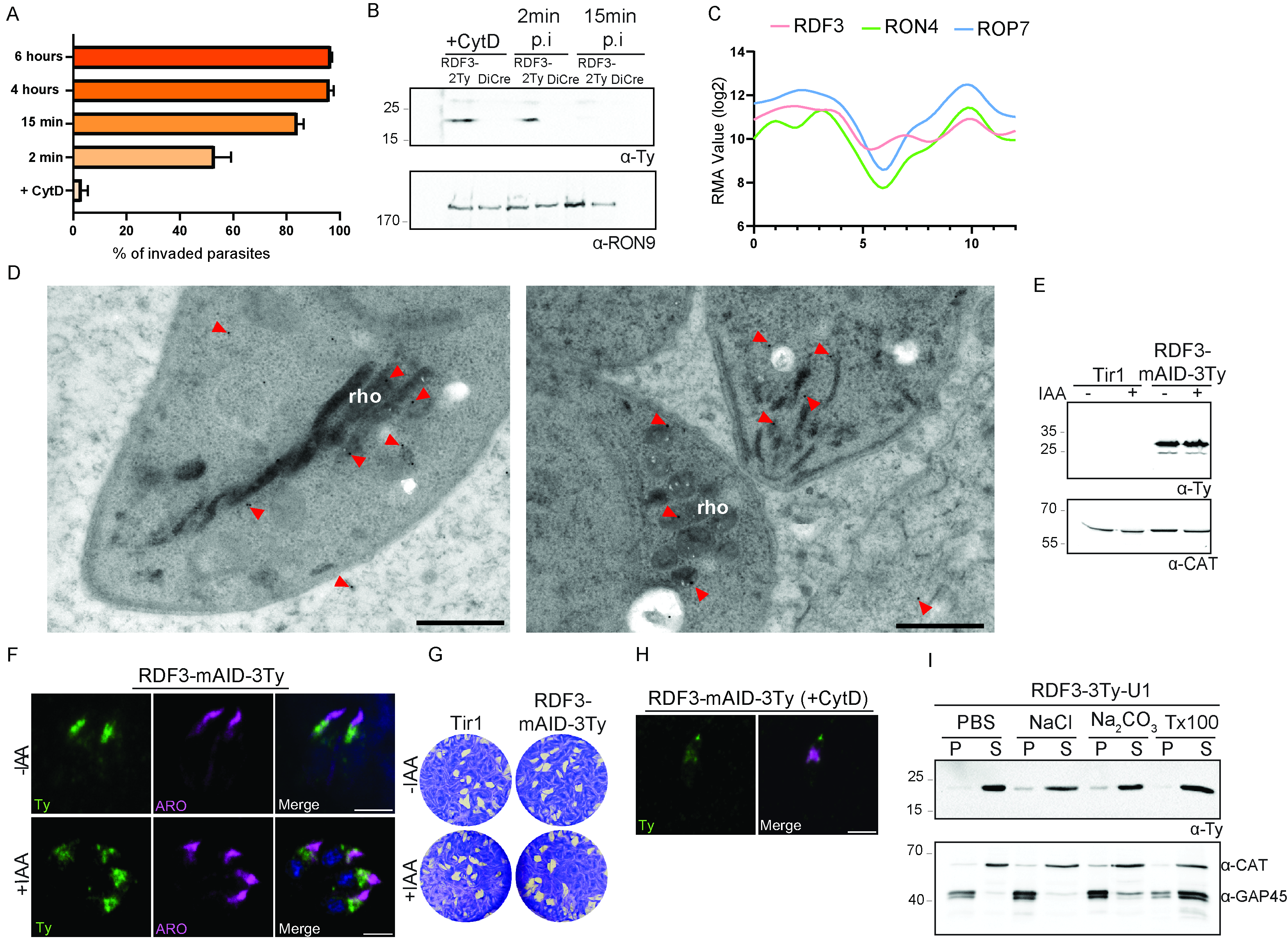

Supplement: S5 Fig — (A) Quantification of invasion of parasites at different time points post-invasion (n = 3 biologically independent experiments). (B) Western blot showing the expression of RDF3-2Ty at different time points. (C) Cell cycle transcriptomic profile of RDF3. Cell cycle was compared to RON4 and ROP7 for control. (D) Immunogold labeling of RDF3-2Ty. Left panel shows longitudinal section through apical part of the parasites, right panel shows transversal section through the bundle of rhoptries. Gold particles location is highlighted with red arrowheads. rho: rhoptries. Scale bar = 500 nm. (E) Western blot showing the absence of down-regulation of RDF3 in presence of IAA. Catalase (CAT) is used as a loading control. (F) Immunofluorescence of intracellular RDF3-mAID-3Ty parasites (±IAA). DAPI (blue). Scale bar = 2 μm. (G) Plaque assay showing that depletion of RDF3 using the mAID system does not affect parasite survival. Parental strain TiR1 was used as a control. Image representative of 3 biologically independent experiments. (H) Immunofluorescence of extracellular RDF3-mAID-3Ty parasites (±IAA) treated with cytochalasin D (+CytD). DAPI (blue). Scale bar = 2 μm. (I) Solubility assay of RDF3-3Ty-U1. P = pellet. S = supernatant. Catalase (cytoplasmic protein) is used as a control soluble in all conditions. GAP45 (anchored to IMC and PM) is used as a control only soluble by Tx-100. Source data are provided as S1 Data. (TIF) [file pbio.3002745.s005.tif]

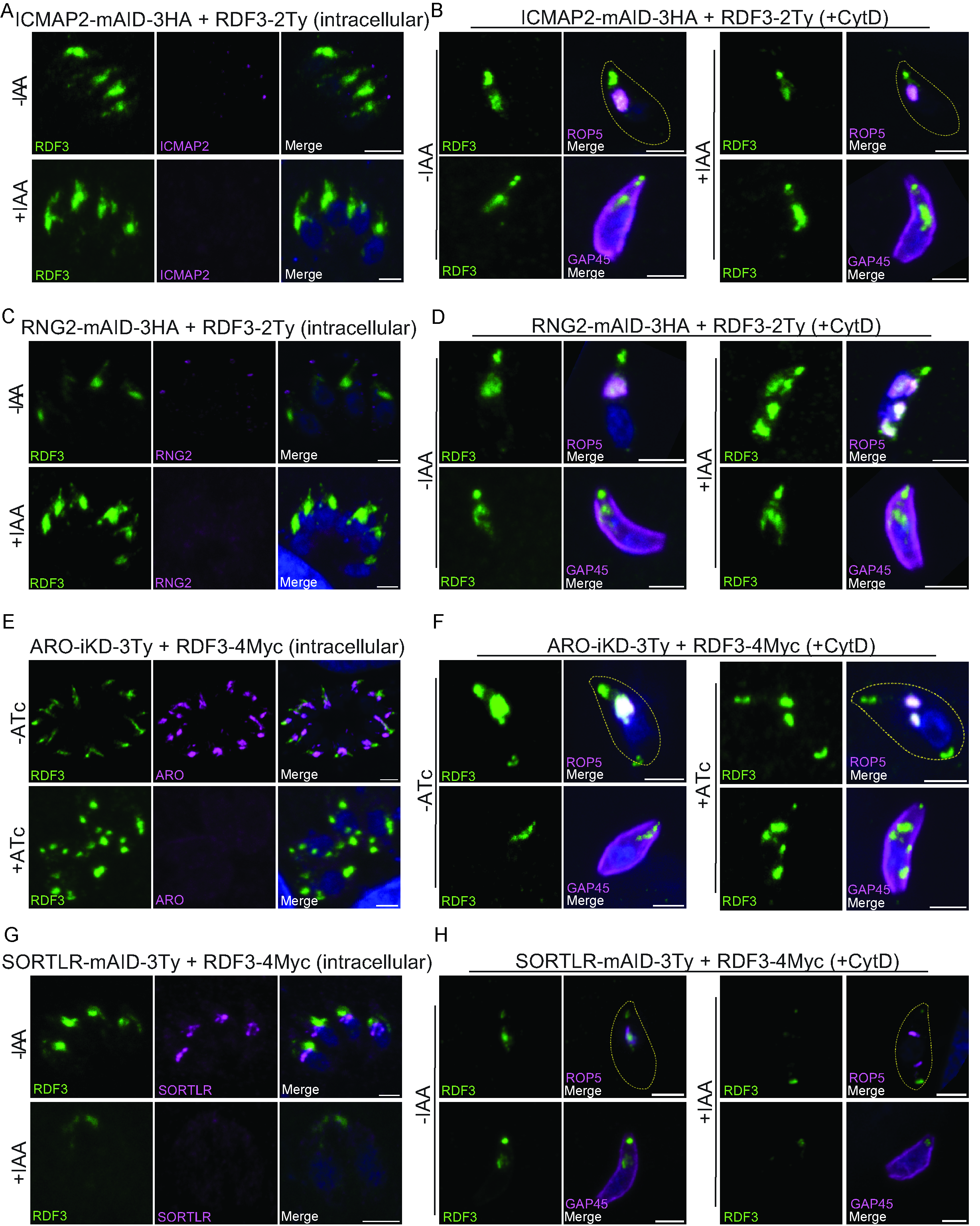

Supplement: S6 Fig — (A) Immunofluorescence showing the localization of RDF3 and ICMAP2 in intracellular parasites (±IAA). DAPI (blue). Scale bar = 2 μm. (B) Immunofluorescence on extracellular parasites (+CytD) using anti-ROP5 and anti-GAP45 antibodies to localize RDF3 in presence or absence of ICMAP2. DAPI (blue). Scale bar = 2 μm. (C) Immunofluorescence showing the localization of RDF3 and RNG2 in intracellular parasites (±IAA). DAPI (blue). Scale bar = 2 μm. (D) Immunofluorescence on extracellular parasites (+CytD) using anti-ROP5 and anti-GAP45 antibodies to localize RDF3 in presence or absence of RNG2. DAPI (blue). Scale bar = 2 μm. (E) Immunofluorescence showing the localization of RDF3 and ARO in intracellular parasites (±ATc). DAPI (blue). Scale bar = 2 μm. (F) Immunofluorescence on extracellular parasites (+CytD) using anti-ROP5 and anti-GAP45 antibodies to localize RDF3 in presence or absence of ARO. DAPI (blue). Scale bar = 2 μm. (G) Immunofluorescence showing the localization of RDF3 and SORTLR in intracellular parasites (±IAA). DAPI (blue). Scale bar = 2 μm. (H) Immunofluorescence on extracellular parasites (+CytD) using anti-ROP5 and anti-GAP45 antibodies to localize RDF3 in presence or absence of SORTLR. DAPI (blue). Scale bar = 2 μm. Source data are provided as S1 Data. (TIF) [file pbio.3002745.s006.tif]

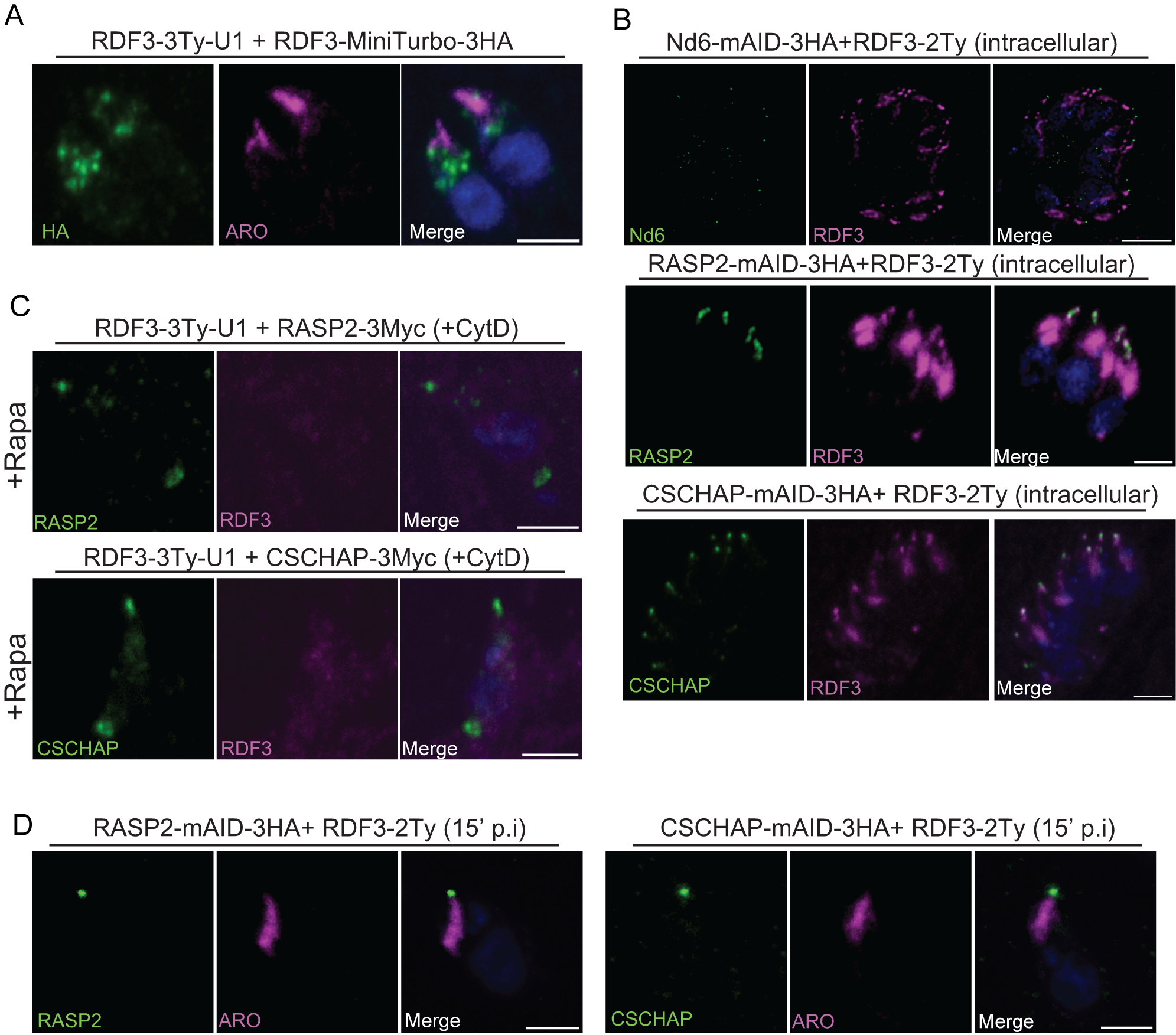

Supplement: S7 Fig — (A) Immunofluorescence showing the mislocalization of RDF3 second copy when tagged with the MiniTurbo cassette. (B) Immunofluorescence to assess RDF3 localization with Nd6, RASP2, and CSCHAP in intracellular parasites. Counter-staining of DNA with DAPI (blue). Scale bar = 2 μm. (C) Immunofluorescence to assess RASP2 and CSCHAP localization in absence of RDF3 in extracellular parasites (+CytD). Counter-staining of DNA with DAPI (blue). Scale bar = 2 μm. Staining observed at the basal pole of the parasite is due to a nonspecific labeling of the anti-Myc rabbit antibody. (D) Immunofluorescence showing the localization of RASP2 and CSCHAP 15 min post-invasion. Counter-staining of DNA with DAPI (blue). Scale bar = 2 μm. Source data are provided as S1 Data. (TIFF) [file pbio.3002745.s007.tiff]

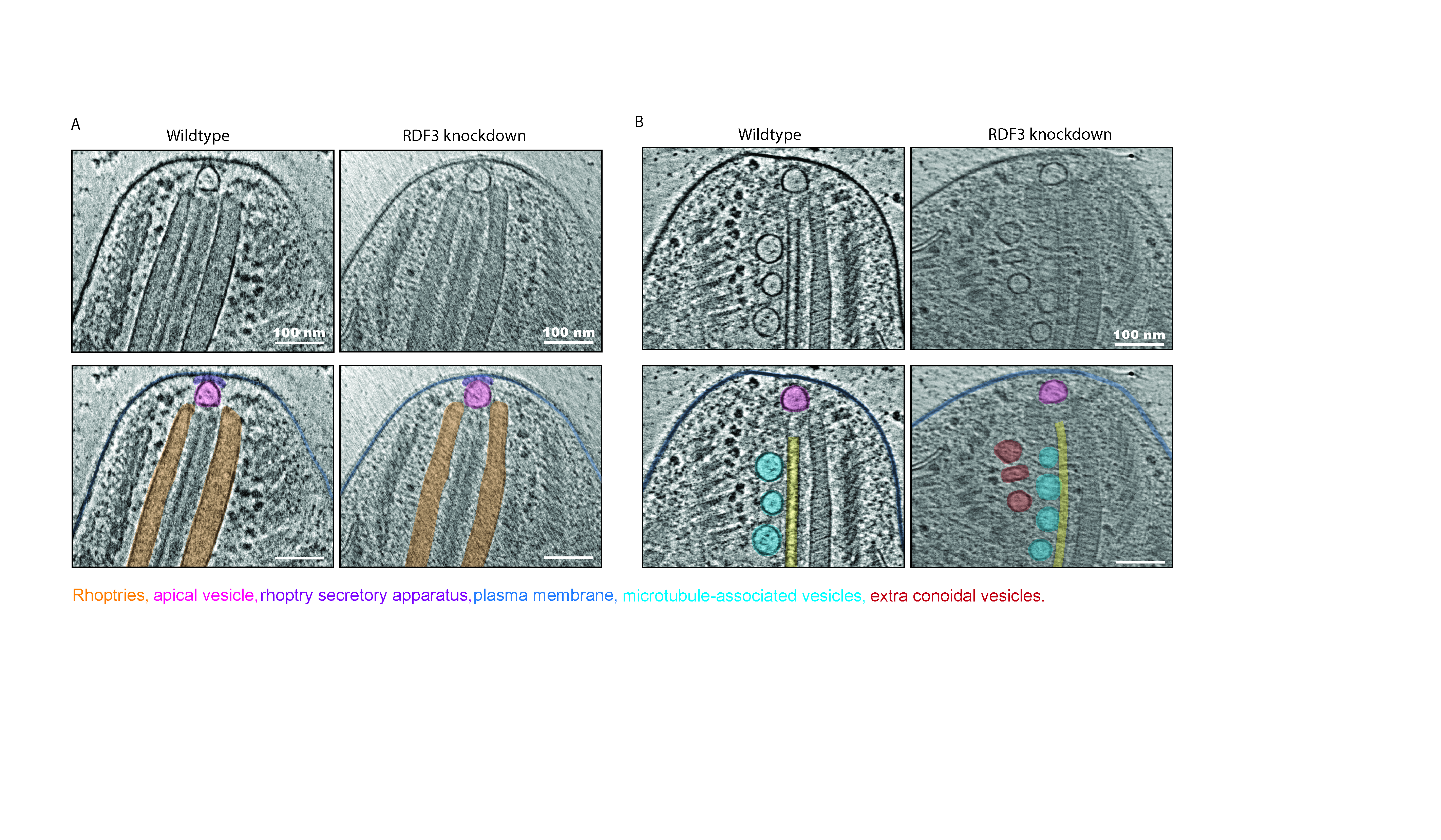

Supplement: S8 Fig — 2D slice of a tomogram through a wild-type (left) and RDF3 knockdown cell (right), with and without color overlay, displaying (A) 2 rhoptries docked at the AV, and (B) the MVs along the ICMT as well as any extra vesicles in the conoid. (TIF) [file pbio.3002745.s008.tif]

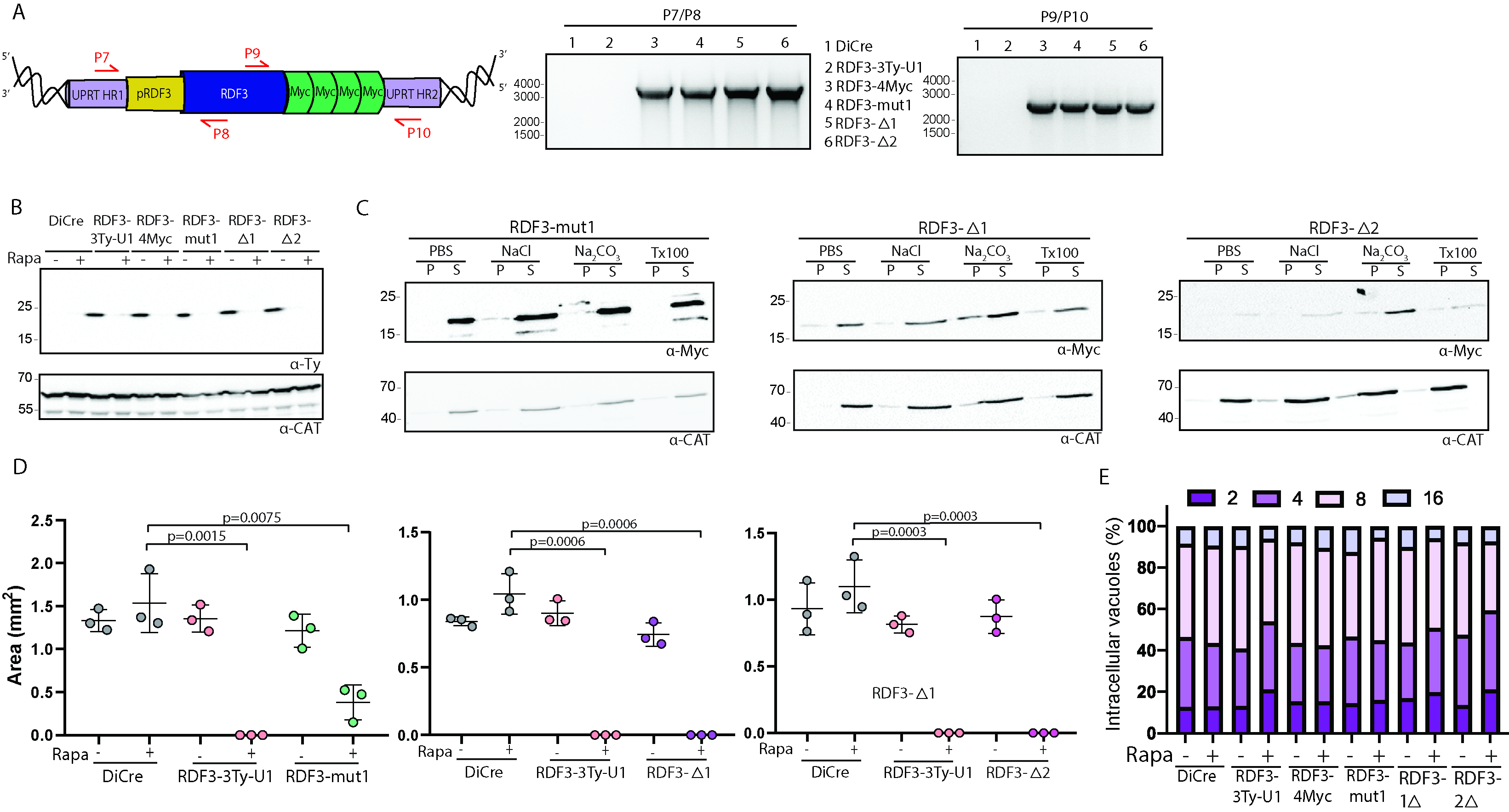

Supplement: S9 Fig — (A) Integration PCR on RDF3 mutants, RDF3-depleted parasites and DiCre parental line performed with primers P7/P8 (5′ integration = 3,300 bp) and P9/P10 (3′ integration = 2,600 bp). (B). Western blot using anti-Ty antibodies showing the regulation of RDF3-3ty-U1 in the complemented mutated strains. Catalase (anti-CAT) is used as a loading control. (C) Solubility assay of RDF3 mutants. P = pellet. S = supernatant. Catalase (cytoplasmic protein) is used as a control soluble in all conditions. (D) Quantification of plaque assays for DiCre, RDF3-3Ty-U1, and the 3 RDF3 mutants’ strain (±Rapa). (Mean ± SD; n = 3 biologically independent experiments.) Statistical significance was assessed by a two-way ANOVA significance with Tukey’s multiple comparison. (E) Intracellular replication assay. Graph representing the number of parasites per vacuole observed at 36 h post-invasion. (Mean ± SD; n = 3 biologically independent experiments.) Source data are provided as S1 Data. (TIF) [file pbio.3002745.s009.tif]
