# Supplementary material for: Toxoplasma gondii rhoptry discharge factor 3 is essential for invasion and microtubule-associated vesicle biogenesis
Source: PLoS Biol. 2024 Aug 13;22(8):e3002745. doi: 10.1371/journal.pbio.3002745 (PMC11343613; doi:10.1371/journal.pbio.3002745)
Supplement: S1 Raw Images — (PDF) [file pbio.3002745.s016.pdf]

Fig 2C

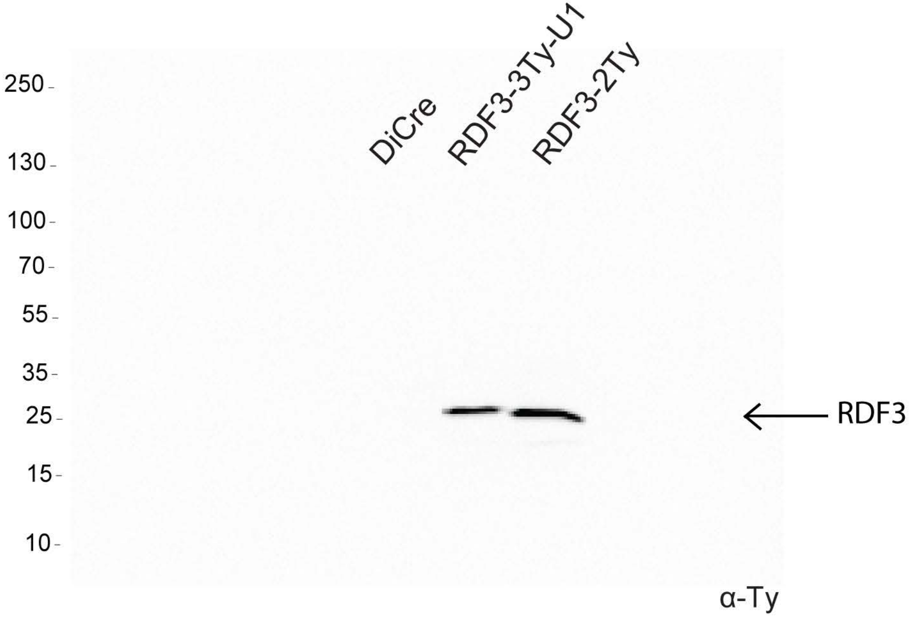
250
130
100
70
55
35
25
15
10
DiCre
RDF3-3Ty-U1
RDF3-2Ty
RDF3
α-Ty

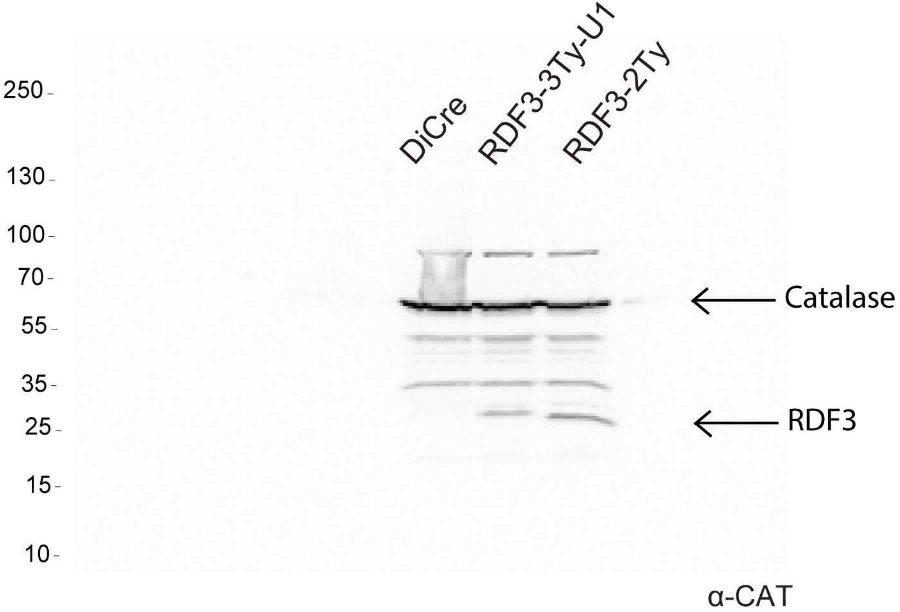
250
130
100
70
55
35
25
15
10
DiCre
RDF3-3Ty-U1
RDF3-2Ty
Catalase
RDF3
α-CAT

Fig 4A

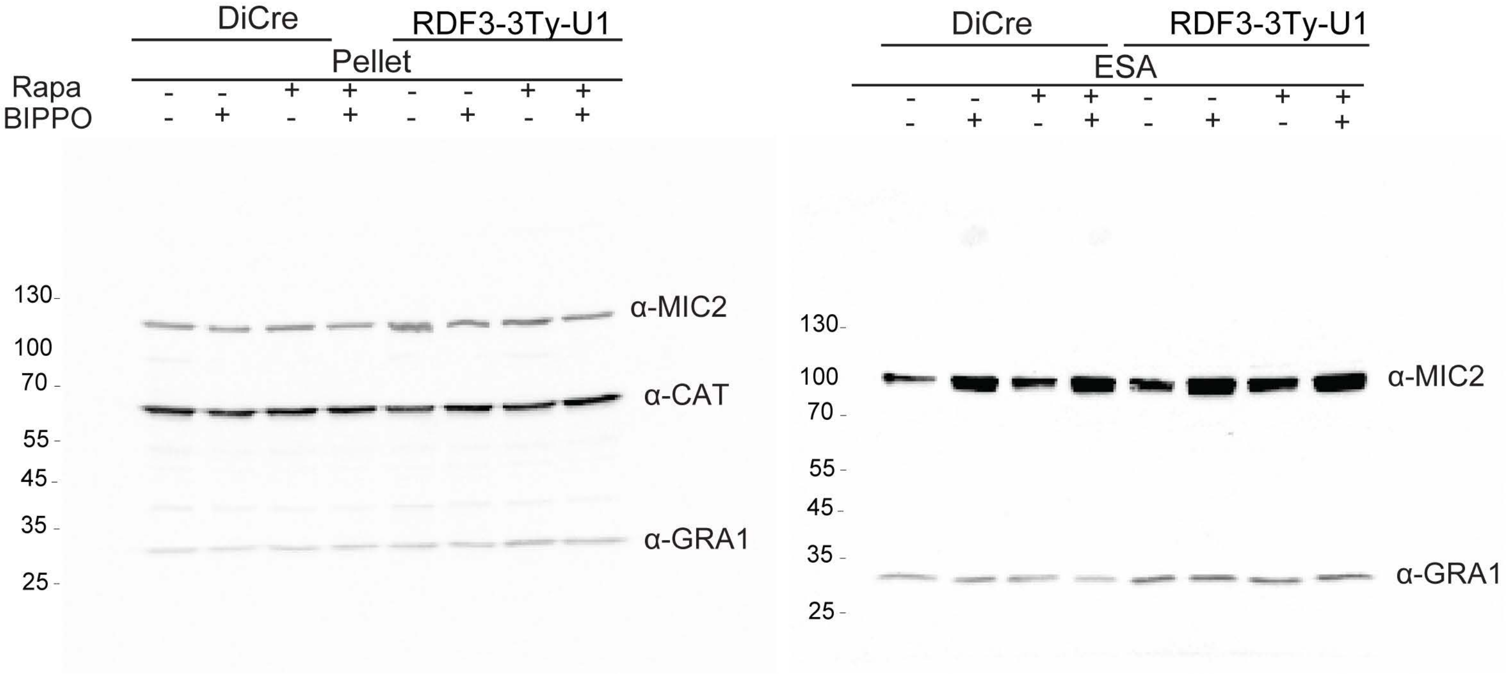
DiCre
RDF3-3Ty-U1
Pellet
Rapa
BIPPO
- - + + - - + +
- + - + - + - +
130
100
70
55
45
35
25
α-MIC2
α-CAT
α-GRA1
DiCre
RDF3-3Ty-U1
ESA
- - + + - - + +
- + - + - + - +
130
100
70
55
45
35
25
α-MIC2
α-GRA1

Fig 5D

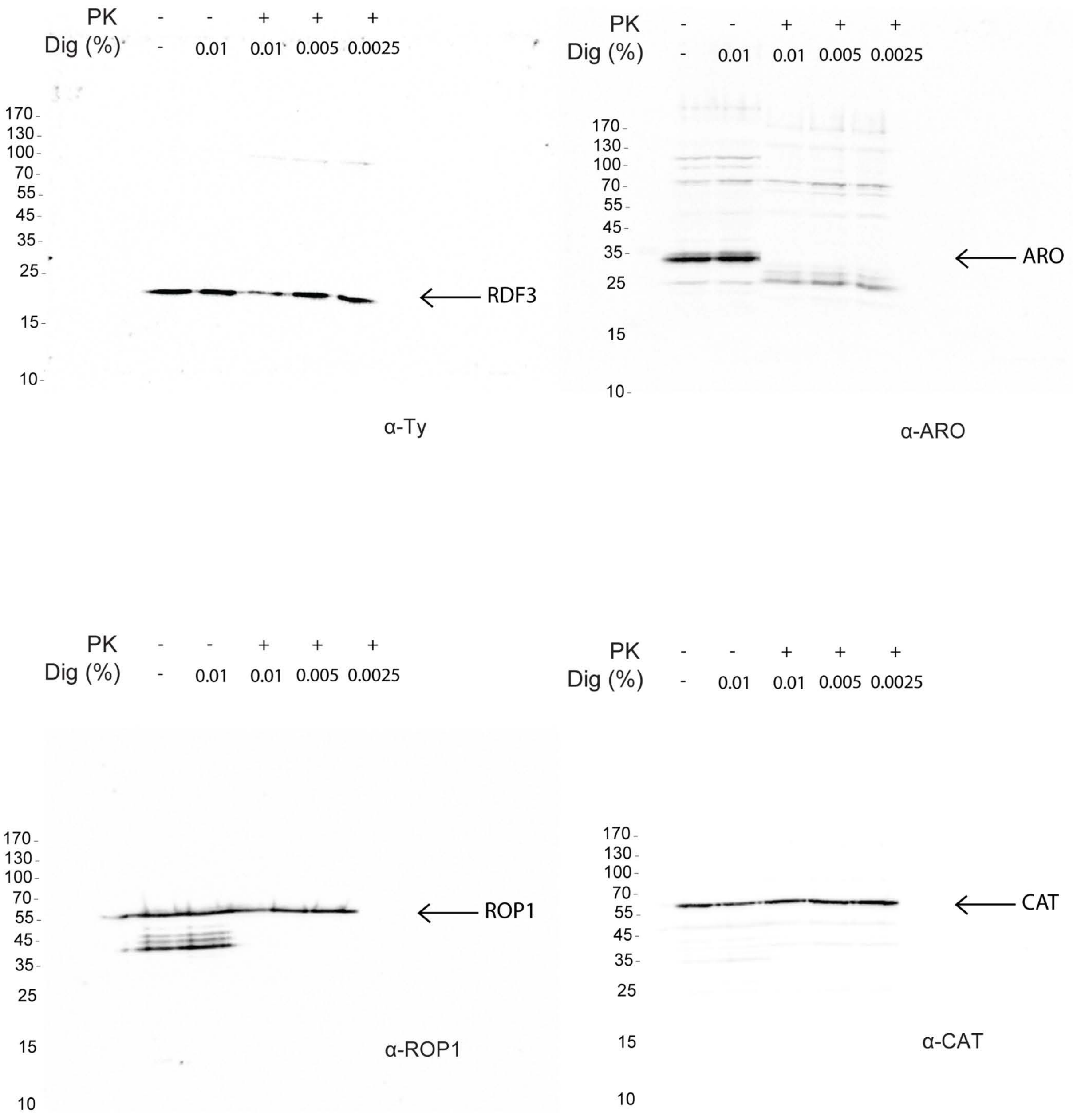
PK - - + + +
Dig (%) - 0.01 0.01 0.005 0.0025
170
130
100
70
55
45
35
25
15
10
RDF3
α-Ty
PK - - + + +
Dig (%) - 0.01 0.01 0.005 0.0025
170
130
100
70
55
45
35
25
15
10
ARO
α-ARO
PK - - + + +
Dig (%) - 0.01 0.01 0.005 0.0025
170
130
100
70
55
45
35
25
15
10
ROP1
α-ROP1
PK - - + + +
Dig (%) - 0.01 0.01 0.005 0.0025
170
130
100
70
55
45
35
25
15
10
CAT
α-CAT

Fig 9B

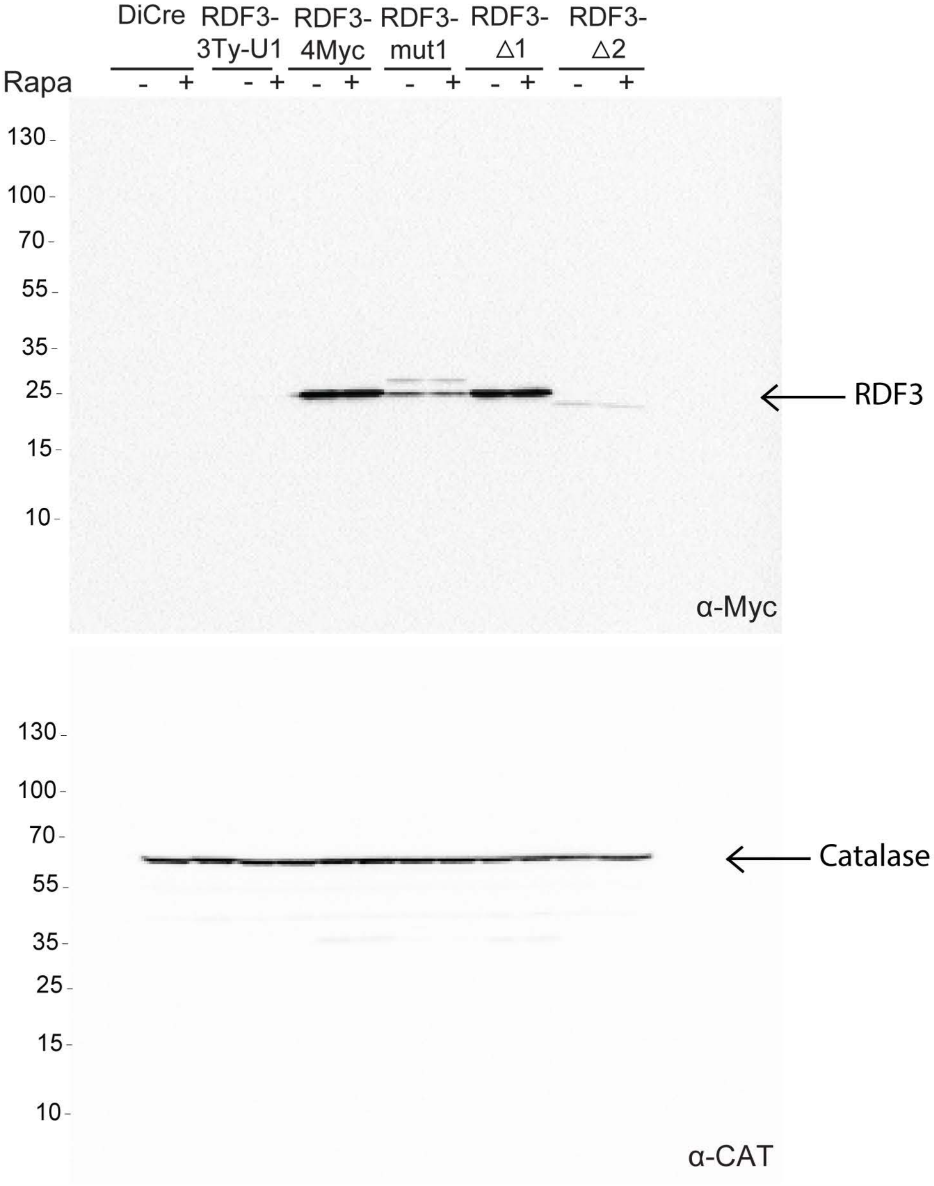
DiCre
RDF3-3Ty-U1
RDF3-4Myc
RDF3-mut1
RDF3-△1
RDF3-△2
Rapa
- + - + - + - + - + - +
130
100
70
55
35
25
15
10
RDF3
α-Myc
130
100
70
55
35
25
15
10
Catalase
α-CAT

S1A Fig

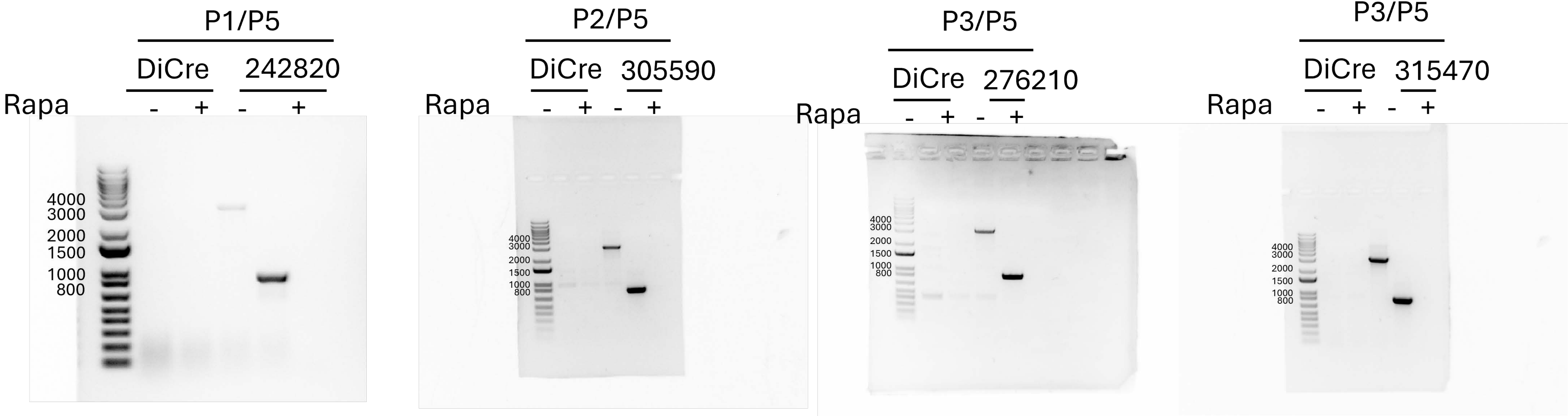

P1/P5
DiCre
242820
Rapa
- + - +
4000
3000
2000
1500
1000
800
P2/P5
DiCre
305590
Rapa
- + - +
4000
3000
2000
1500
1000
800
P3/P5
DiCre
276210
Rapa
- + - +
4000
3000
2000
1500
1000
800
P3/P5
DiCre
315470
Rapa
- + - +
4000
3000
2000
1500
1000
800

S1B Fig

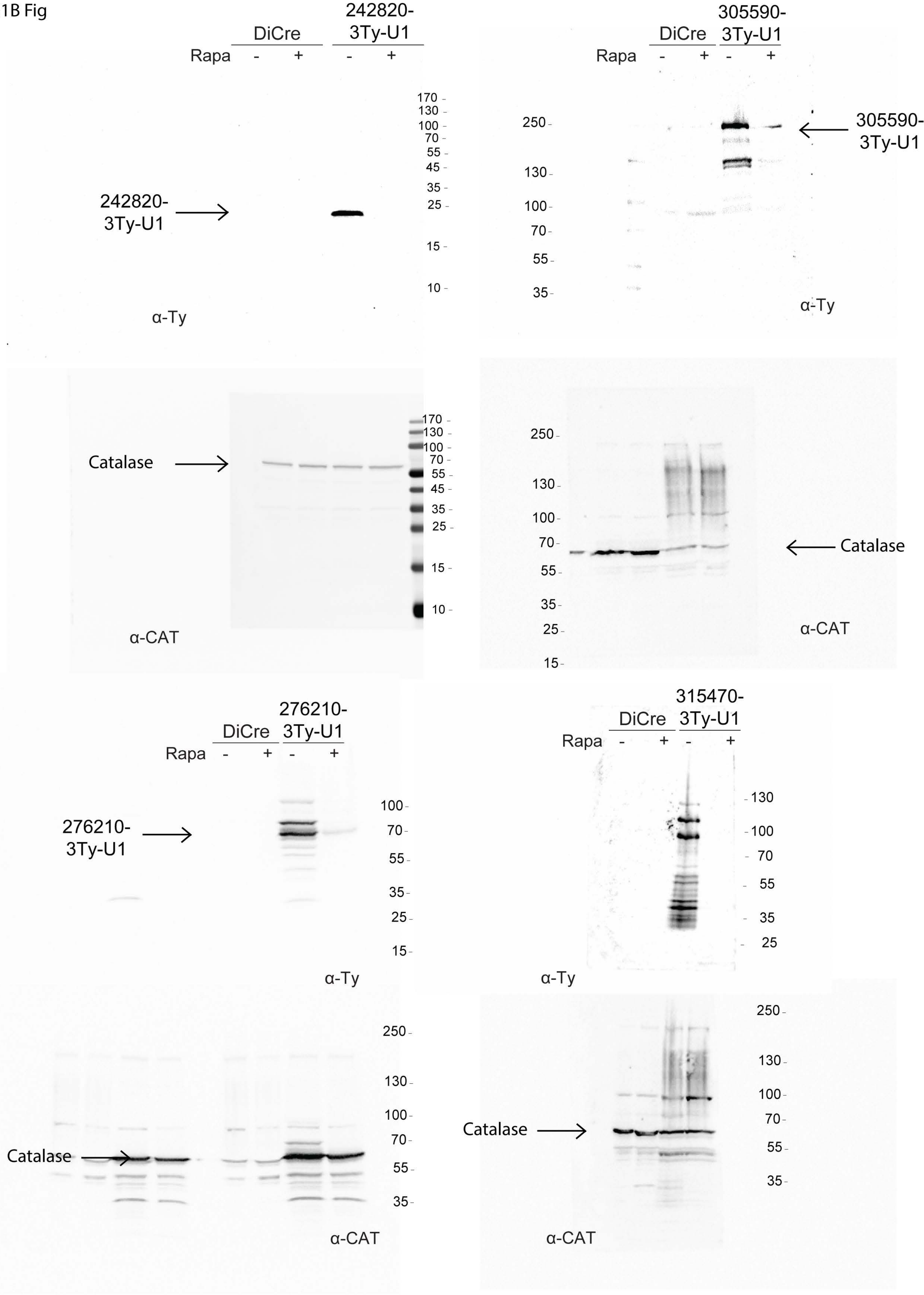
DiCre
242820-
3Ty-U1
Rapa
-
+
-
+
170
130
100
70
55
45
35
25
15
10
242820-
3Ty-U1
α-Ty
DiCre
305590-
3Ty-U1
Rapa
250
130
100
70
55
35
305590-
3Ty-U1
α-Ty
Catalase
170
130
100
70
55
45
35
25
15
10
α-CAT
250
130
100
70
55
35
25
15
Catalase
α-CAT
DiCre
276210-
3Ty-U1
Rapa
100
70
55
35
25
15
276210-
3Ty-U1
α-Ty
DiCre
315470-
3Ty-U1
Rapa
130
100
70
55
35
25
α-Ty
250
130
100
70
55
35
Catalase
α-CAT
250
130
100
70
55
35
Catalase
α-CAT

S2A Fig

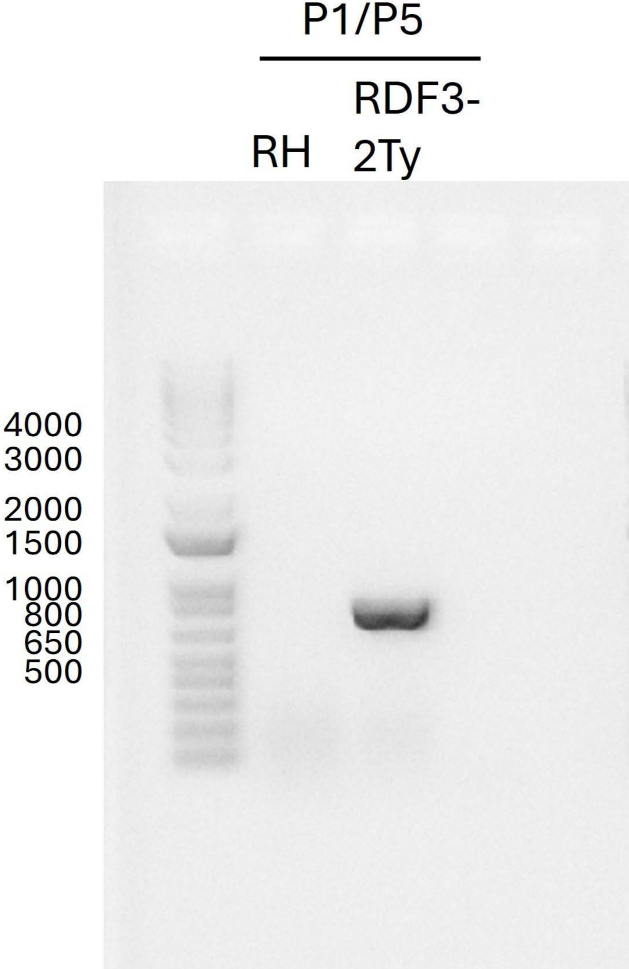

S3A Fig

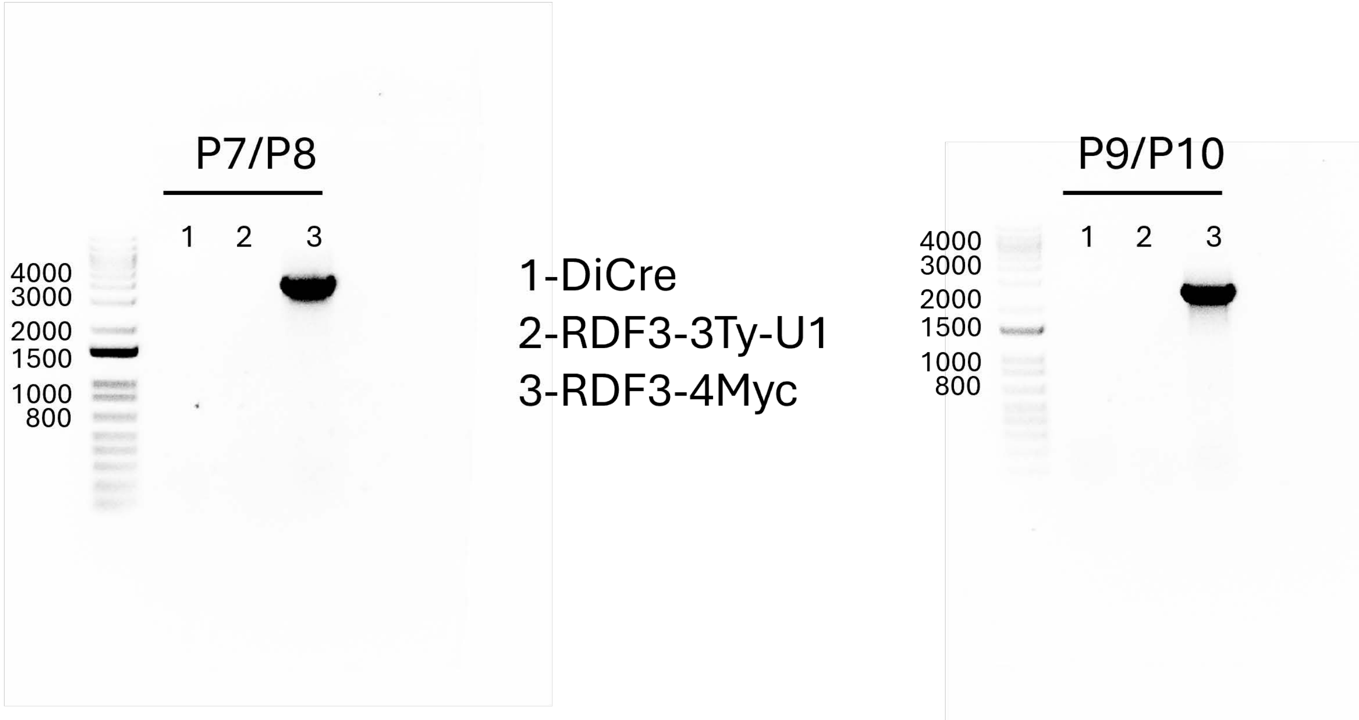
P7/P8
1 2 3
4000
3000
2000
1500
1000
800
1-DiCre
2-RDF3-3Ty-U1
3-RDF3-4Myc
P9/P10
1 2 3
4000
3000
2000
1500
1000
800

S3B Fig

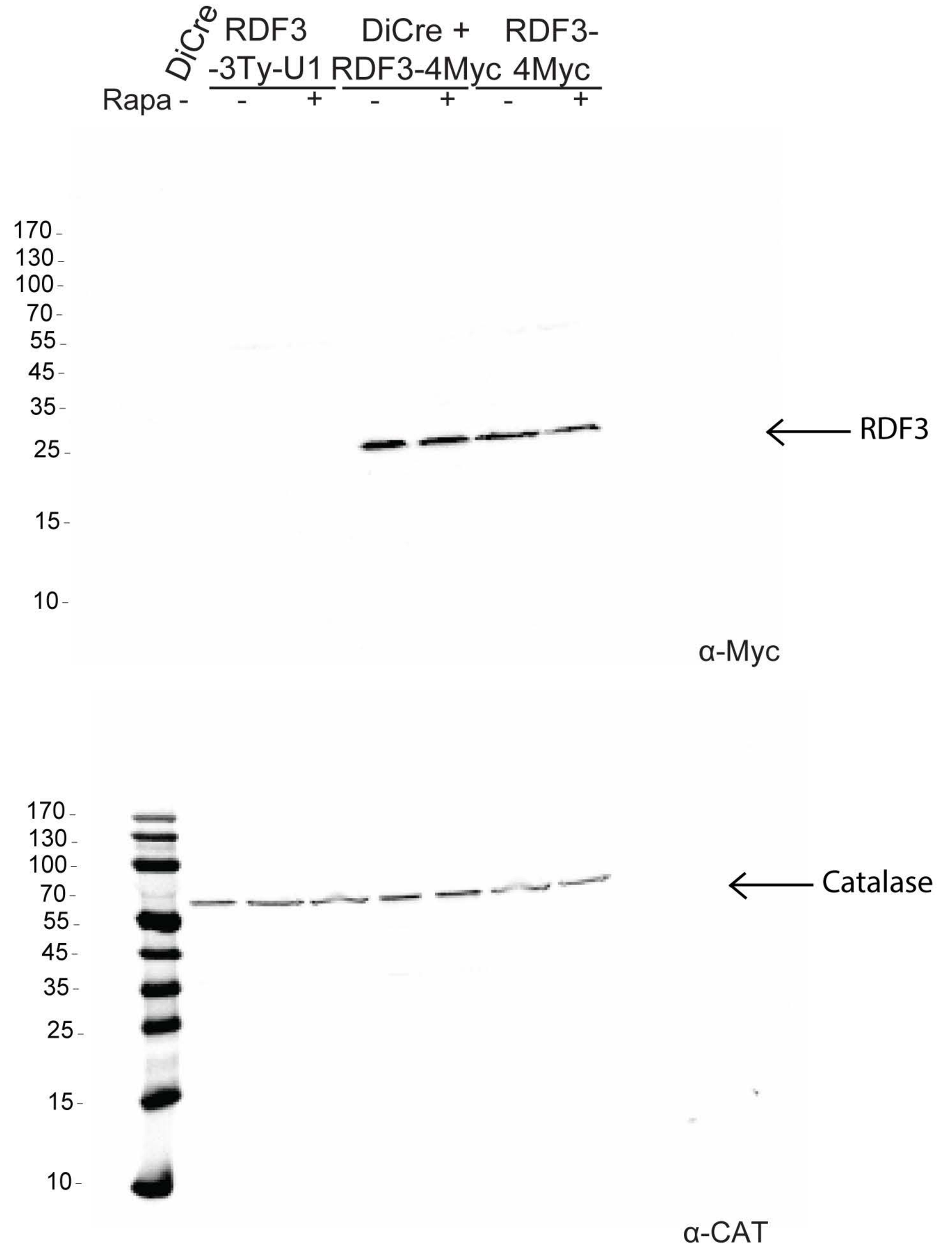

DiCre
RDF3 -3Ty-U1
DiCre + RDF3-4Myc
RDF3- 4Myc
Rapa - - + - + - +
170
130
100
70
55
45
35
25
15
10
RDF3
α-Myc
170
130
100
70
55
45
35
25
15
10
Catalase
α-CAT

B

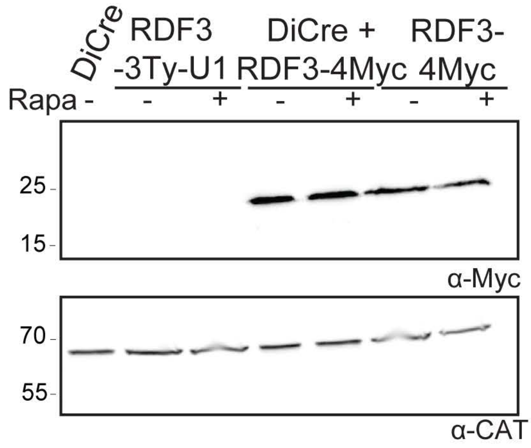

DiCre
RDF3 -3Ty-U1
DiCre + RDF3-4Myc
RDF3- 4Myc
Rapa - - + - + - +
25
15
α-Myc
70
55
α-CAT

S4B Fig

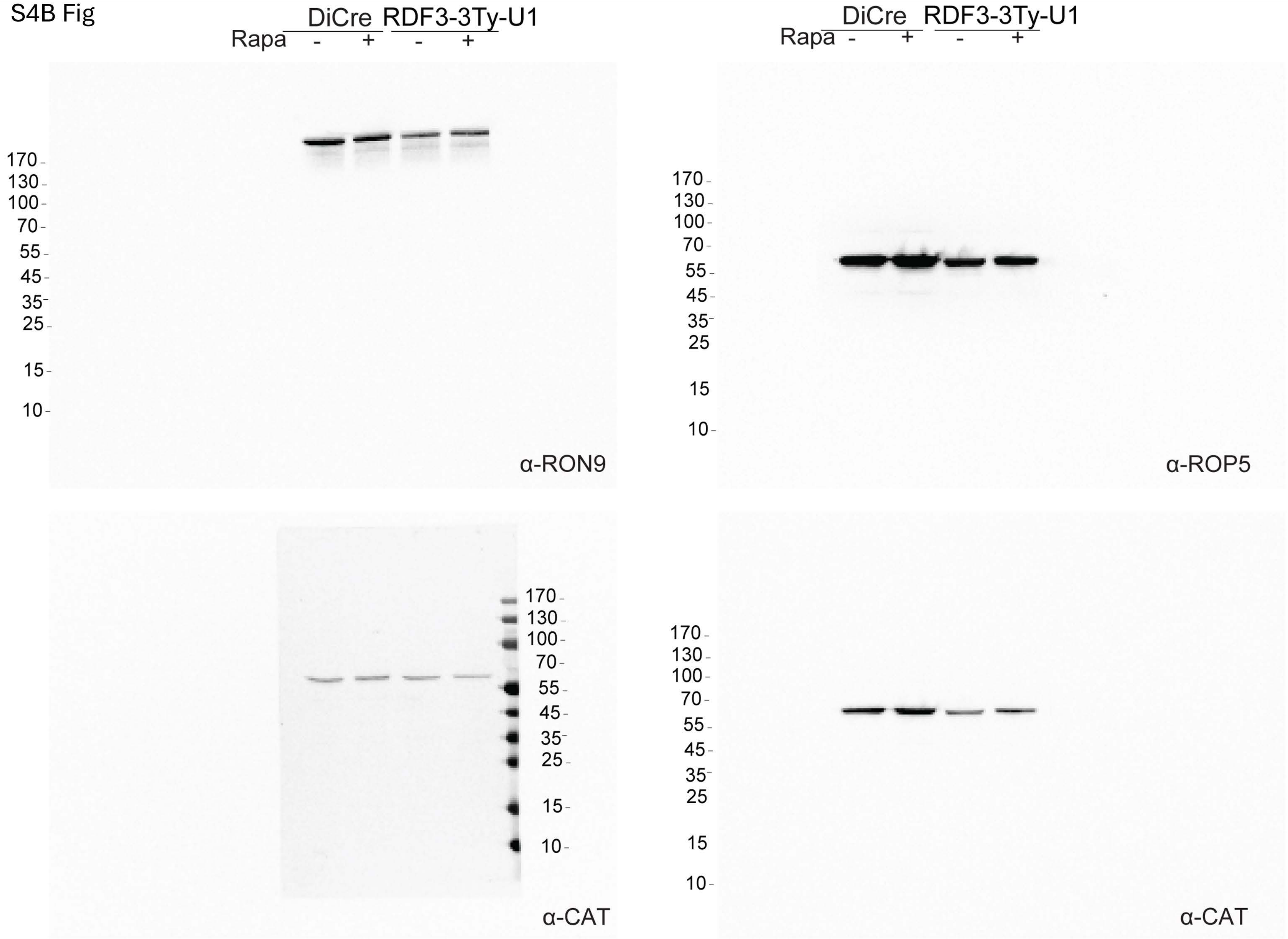

DiCre
RDF3-3Ty-U1
Rapa
-
+
-
+
170
130
100
70
55
45
35
25
15
10
α-RON9
α-ROP5
α-CAT
α-CAT

S5B Fig

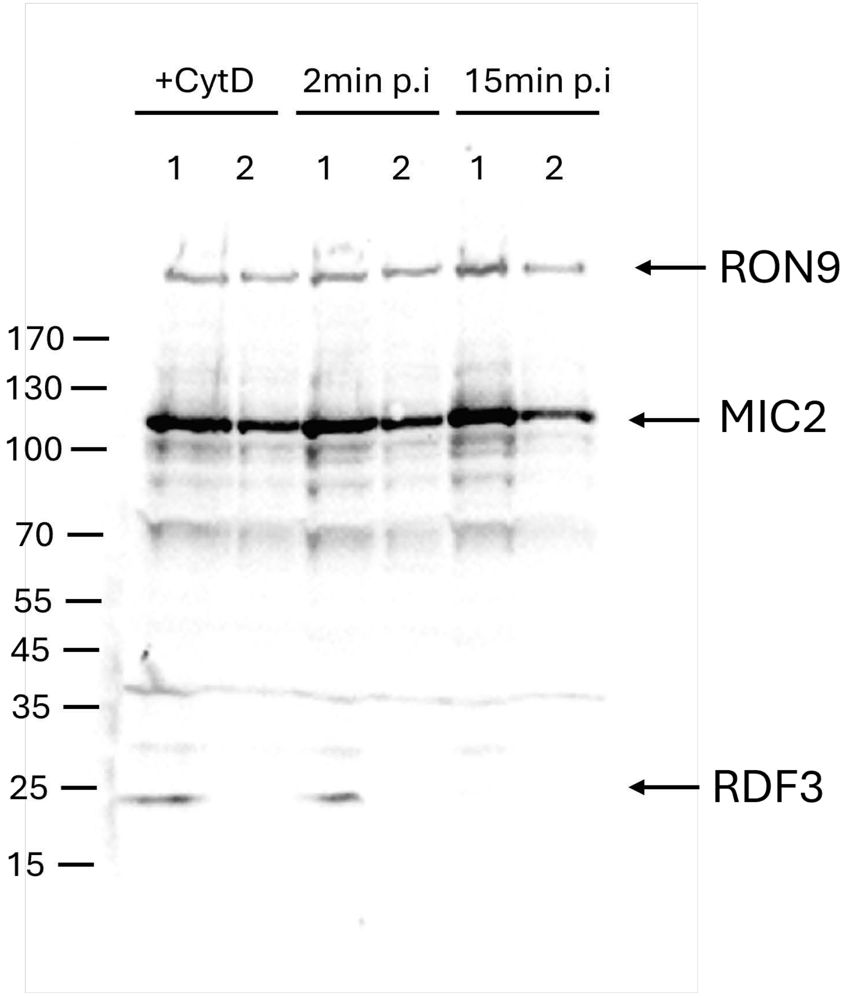

1. RDF3-2Ty
2. DiCre

MIC2 was used as a control but was not added to the final panel. No defect was observed.

S5E Fig

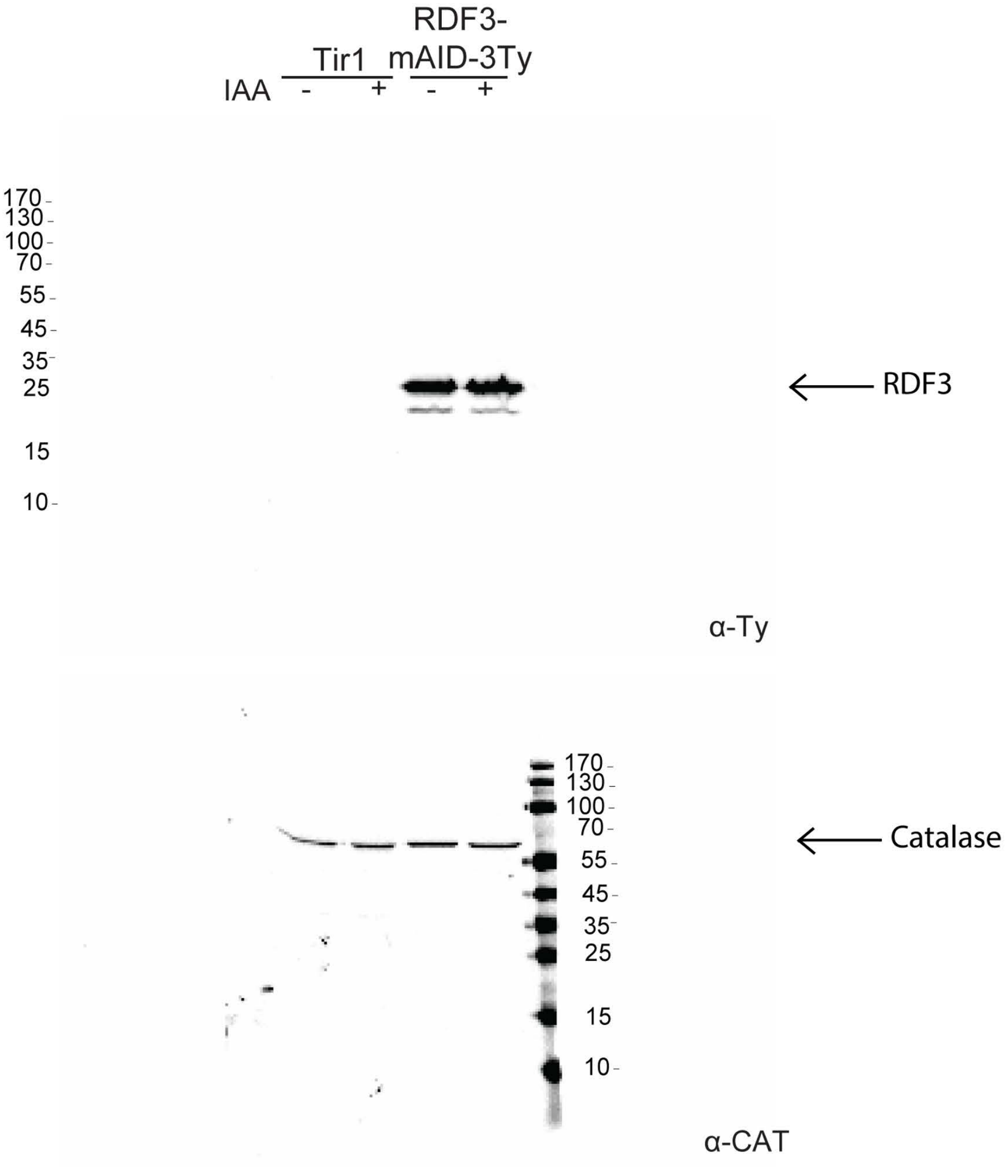
RDF3-
Tir1
mAID-3Ty
IAA - + - +
170
130
100
70
55
45
35
25
15
10
RDF3
α-Ty
170
130
100
70
55
45
35
25
15
10
Catalase
α-CAT

S5I Fig

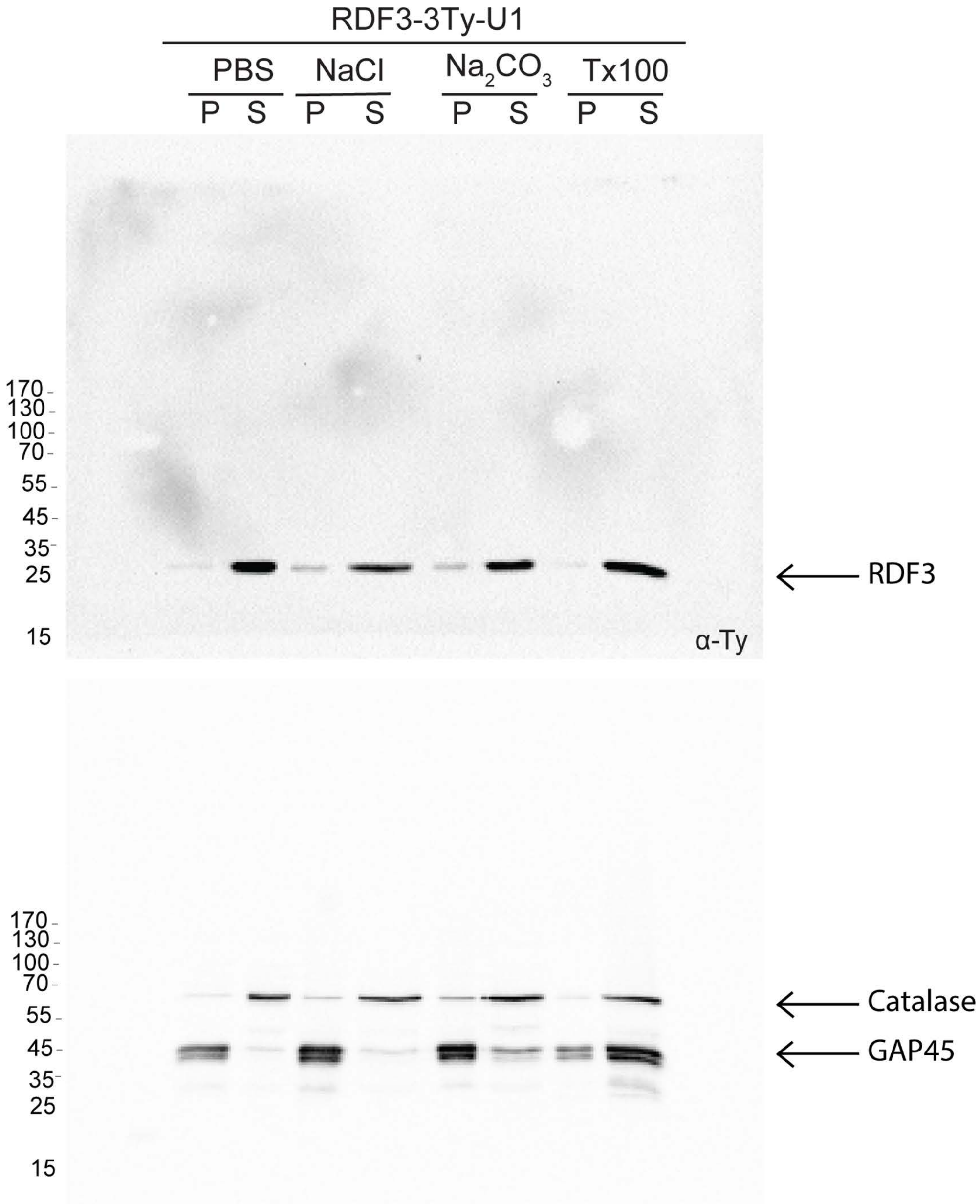

RDF3-3Ty-U1
PBS
NaCl
Na_2CO_3
Tx100
P S P S P S P S
170
130
100
70
55
45
35
25
15
RDF3
α-Ty
170
130
100
70
55
45
35
25
15
Catalase
GAP45

S9A Fig

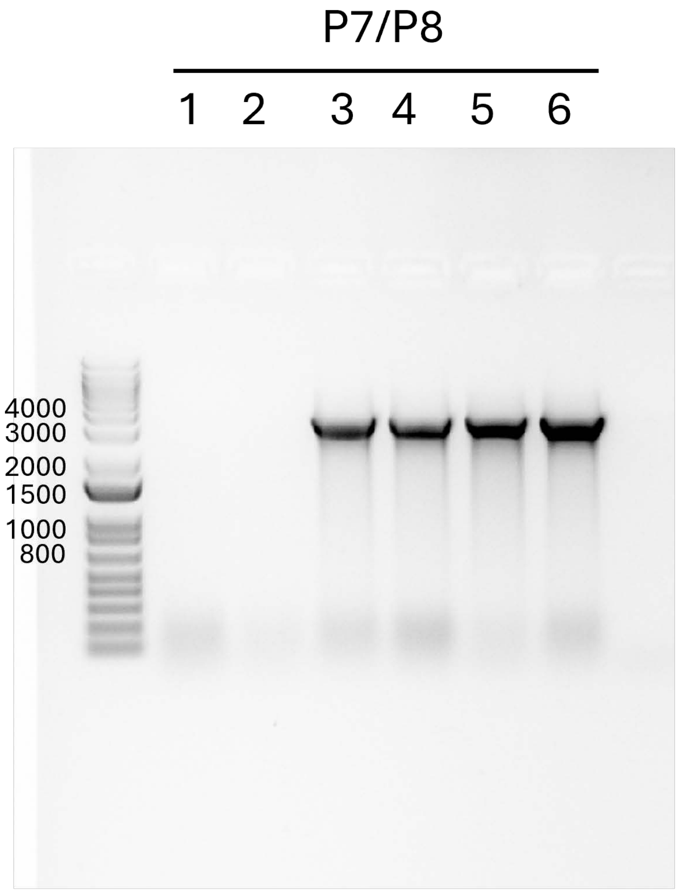

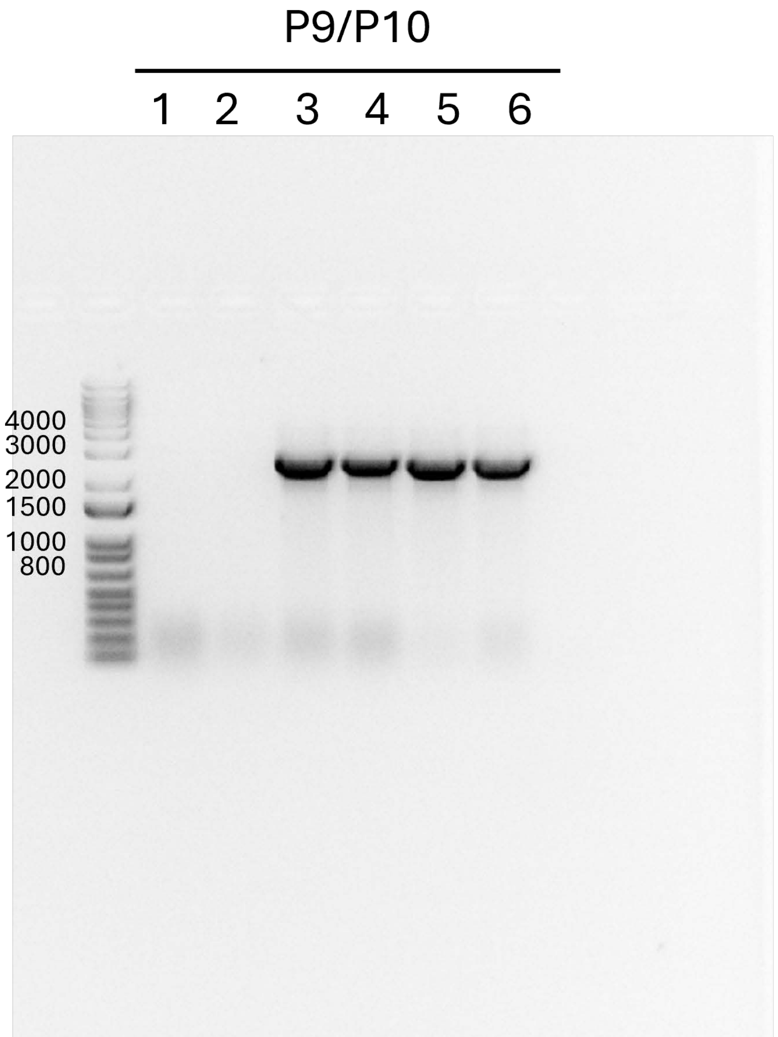

1 DiCre
2 RDF3-3y-U1
3 RDF3-4Myc
4 RDF3-Mut1
5 RDF3-Δ1
6 RDF3-Δ2

S9B Fig

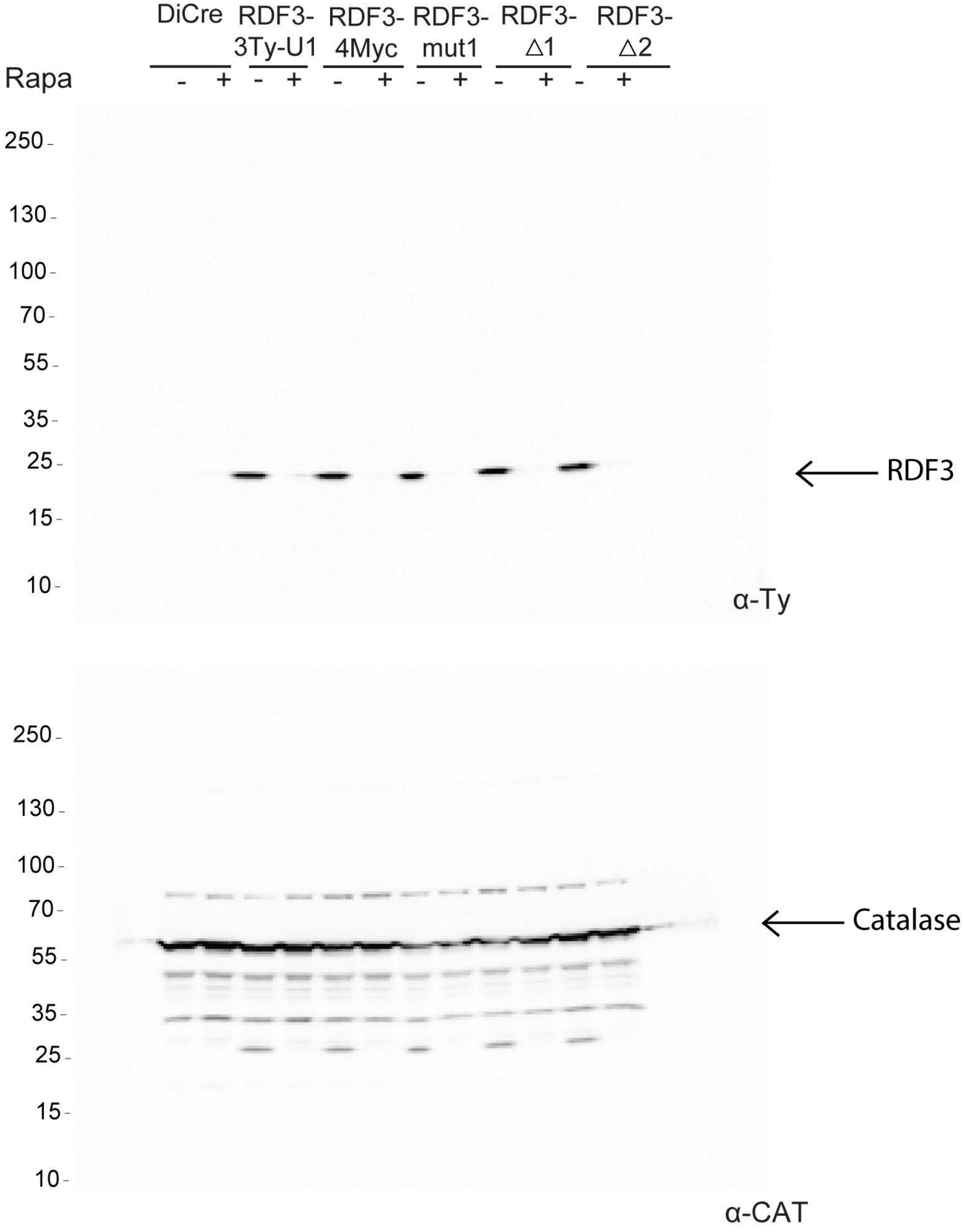
DiCre
RDF3-3Ty-U1
RDF3-4Myc
RDF3-mut1
RDF3-△1
RDF3-△2
Rapa
- + - + - + - + - + - +
250
130
100
70
55
35
25
15
10
RDF3
α-Ty
250
130
100
70
55
35
25
15
10
Catalase
α-CAT

S9C Fig

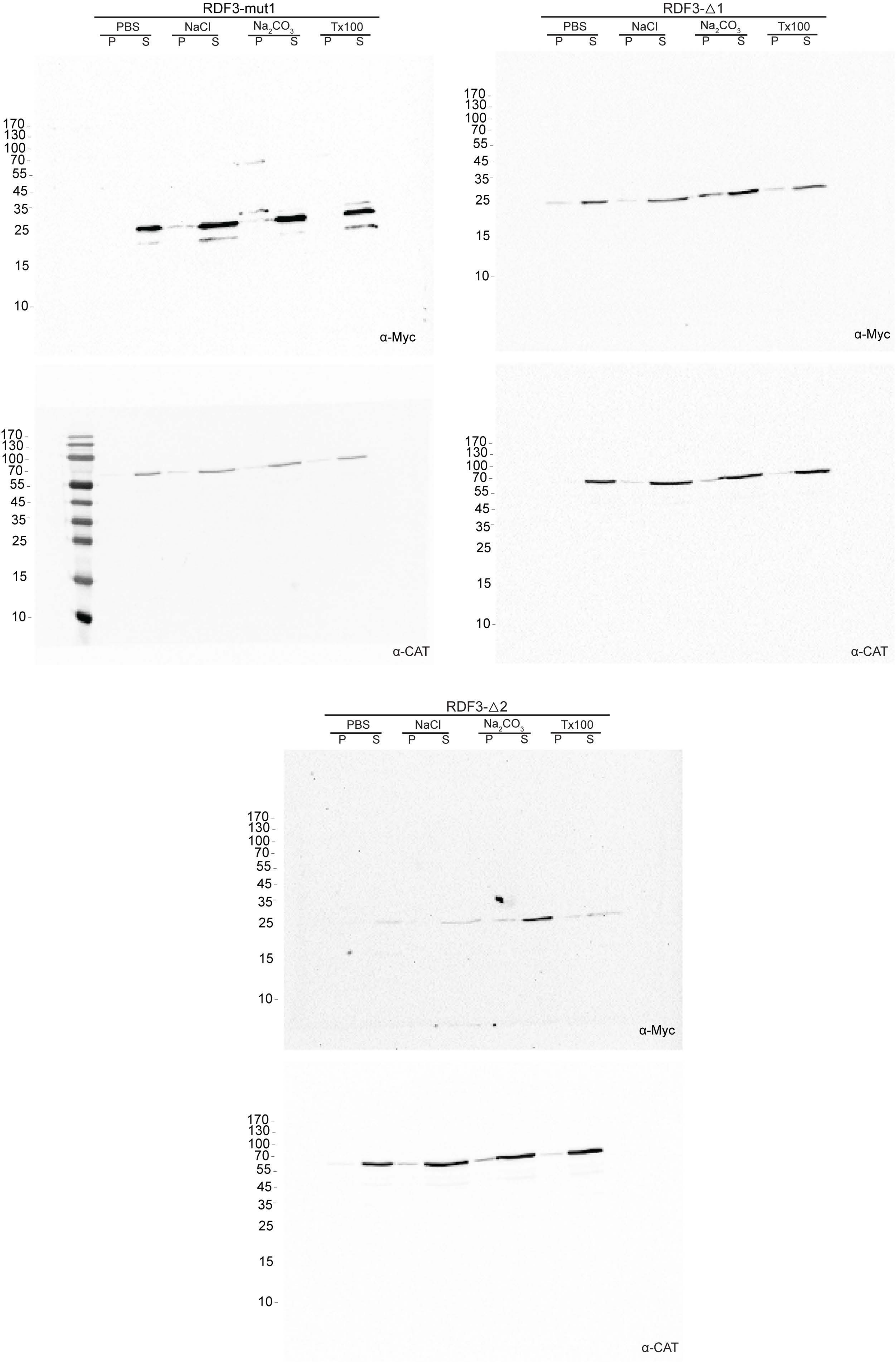
RDF3-mut1
PBS
NaCl
Na_2CO_3
Tx100
P S P S P S P S
170
130
100
70
55
45
35
25
15
10
α-Myc
α-CAT
RDF3-△1
PBS
NaCl
Na_2CO_3
Tx100
P S P S P S P S
α-Myc
α-CAT
RDF3-△2
PBS
NaCl
Na_2CO_3
Tx100
P S P S P S P S
α-Myc
α-CAT
